# Time Trends in Incidence and Mortality of Acute Myocardial Infarction, and All-Cause Mortality following a Cardiovascular Prevention Program in Sweden

**DOI:** 10.1371/journal.pone.0140201

**Published:** 2015-11-18

**Authors:** Gunilla Journath, Niklas Hammar, Stig Elofsson, Anette Linnersjö, Max Vikström, Göran Walldius, Ingvar Krakau, Peter Lindgren, Ulf de Faire, Mai-Lis Hellénius

**Affiliations:** 1 Unit of Cardiology, Department of Medicine, Karolinska Institutet, Stockholm, Sweden; 2 Division of Epidemiology, Institute of Environmental Medicine, Karolinska Institutet, Stockholm, Sweden; 3 AstraZeneca R&D, Mölndal, Sweden; 4 Department of Social Work, Stockholm University, Stockholm, Sweden; 5 Centre for Occupational and Environmental Medicine, Stockholm County Council, Stockholm, Sweden; 6 Division of Cardiovascular Epidemiology, Institute of Environmental Medicine, Karolinska Institutet, Stockholm, Sweden; 7 Unit of Clinical Epidemiology, Department of Medicine, Karolinska Institutet, Stockholm, Sweden; 8 Medical Management Center, MMC, Department of Learning, Information, Management and Ethics, Karolinska Institutet, Stockholm, Sweden; 9 The Swedish Institute for Health Economics, Stockholm, Sweden; Mayo Clinic, UNITED STATES

## Abstract

**Background:**

In 1988, a cardiovascular prevention program which combined an individual and a population-based strategy was launched within primary health-care in Sollentuna, a municipality in Stockholm County. The aim of this study was to investigate time trends in the incidence of and mortality from acute myocardial infarction and all-cause mortality in Sollentuna compared with the rest of Stockholm County during a period of two decades following the implementation of a cardiovascular prevention program.

**Materials and Methods:**

The average population in Sollentuna was 56,589 (49% men) and in Stockholm County (Sollentuna included) 1,795,504 (49% men) during the study period of 1987–2010. Cases of hospitalized acute myocardial infarction and death were obtained for the population of Sollentuna and the rest of Stockholm County using national registries of hospital discharges and deaths. Acute myocardial infarction incidence and mortality were estimated using the average population of Sollentuna and Stockholm in 1987–2010.

**Results:**

During the observation period, the incidence of acute myocardial infarction decreased more in Sollentuna compared with the rest of Stockholm County in women (-22% vs. -7%; for difference in slope <0.05). There was a trend towards a greater decline in Sollentuna compared to the rest of Stockholm County in the incidence of acute myocardial infarction (in men), acute myocardial mortality, and all-cause mortality but the differences were not significant.

**Conclusion:**

During a period of steep decline in acute myocardial infarction incidence and mortality in Stockholm County the municipality of Sollentuna showed a stronger trend in women possibly compatible with favorable influence of a cardiovascular prevention program.

**Trial Registration:**

ClinicalTrials.gov NCT02212145

## Introduction

The prevention of cardiovascular disease (CVD) is a matter of great concern, even though coronary heart disease (CHD) has declined substantially in most industrialized countries during the last few decades [1]. The leading cause of death in Sweden and most European countries is still CVD [2,3]. Investigations from several countries indicate that reductions in major CVD risk factors account for the large decline in CHD [4–7]. CHD mortality has decreased by around two-thirds in all age groups in Sweden in recent decades [8]. In Iceland, a 25-year follow-up of a comprehensive CVD prevention program showed a decline in CHD mortality of 80% [7]. This decline in CHD mortality was similar to that in other western populations such as Europe [9, 10] and the US [11] as a result of risk factor changes in the population and improved prevention of CVD in the health care [12].

A few CVD prevention programs in primary care have been performed in Sweden [13–16]. Most of these programs lack evaluations of the long-term effects on acute myocardial infarction (AMI) incidence and mortality, as well as all-cause mortality. However, these programs have shown improvements in lifestyle, as well as reductions in CVD risk factors [17–18]. One, long-term follow-up study of a CVD primary and secondary prevention program in the southern part of Sweden (Habo) reported a larger decrease in ischemic heart disease (IHD) mortality compared with the entire country of Sweden [17]. In 1988, a CVD prevention program which combined an individual and a population-oriented strategy, was started in primary health care in Sollentuna, a municipality in Stockholm County. The design of this prevention program has been published elsewhere [19].

The aim of this study was to investigate time trends in the incidence of and mortality from acute myocardial infarction and all-cause mortality in Sollentuna compared with the rest of Stockholm County during a period of two decades following the implementation of a cardiovascular prevention program.

## Materials and Methods

### Study population

All individuals living in Stockholm County on January 1, 1987 to December 31, 2010 were included in this retrospective, observational register study. Separate analyses were performed for the population of Sollentuna Municipality (Sollentuna) and Stockholm County excluding Sollentuna (Stockholm). The average population per year was 56,589 (49% men) in Sollentuna and 1,795,504 (49% men) in Stockholm County (including Sollentuna).

### Study setting

There were some differences in characteristics between the populations of Sollentuna and Stockholm County in 1990 and 2010. In Sollentuna compared with Stockholm County, the employment rate was higher, a larger proportion were married, a lower proportion were foreign born, and education and wages were generally higher among men and women in both 1990 and 2010 (Table 1).

**Table 1:** Characteristics of the population who lived in Sollentuna, and Stockholm in 1990 and 2010.

	1990	1990	2010	2010
	Sollentuna Municipality	Stockholm County	Sollentuna Municipality	Stockholm County
Population (n)	51 377	1 641 669	64 630	2 054 343
Men, n (%)	25 304 (49.2)***	796 226 (48.5)	32 159 (49.8)	1 016 200 (49.5)
Average age men (years)	35.2	36.9	37.1	37.8
Average age women (years)	37.1	40.0	39.2	40.0
Working men 16–64 years, n (%)	14301 (83.3)***	442932 (82.1)	15194 (74.9)***	500077 (73.5)
Working women 16–64 years, n (%)	14433 (83.5)***	444251 (82.2)	14989 (74.4)***	485037 (72.1)
Married men, n (%)	10597 (41.8)***	298422 (37.4)	11929 (37.1)***	32823 (32.3)
Married women, n (%)	10719 (41.1)***	300423 (35.5)	11963 (36.8)***	327965 (31.6)
Foreign-born men, n (%)	3475 (13.7)	120812 (15.2)***	6124 (19.0)	210834 (20.7)***
Foreign-born women, n (%)	3752 (14.4)	131090 (15.5)***	6497 (20.0)	224600 (21.6)***
Mean income x1,000 SEK	195.1¤	177.0¤	364.3	317.1
Mean income x1,000 SEK	122.8¤	118.2¤	259.6	234.5
Low income, men, n (%) §	NA	NA	3014 (16.4)	123458 (19.7)***
Low income, women, n (%) §	NA	NA	3322 (18.1)	132998 (21.4)***
Medium income, men, n (%) §	NA	NA	7133 (38.9)	281033 (45.0)***
Medium income, women, n (%) §	NA	NA	9942 (54.1)	357949 (57.5)***
High income, men, n (%) §	NA	NA	8192 (44.7)***	222370 (35.5)
High income, women, n (%) §	NA	NA	5121 (27.9)***	131480 (21.1)
Education <10 years, men, n (%)	4793 (25.5)	172278 (29)***	4190 (18.2)	149113 (19.6)***
Education <10 years, women n (%)	5227 (27.3)	185042 (29.8)***	3567 (15.5)	126749 (16.6)***
Education 10–12 years, men, n, (%)	8087 (43.0)	261449 (43.4)	8500 (37.0)	301532 (39.5)***
Education10-12 years, women, n (%)	7932 (41.5)	270749 (44)***	8205 (35.7)	288129 (37.8)***
Education >12 years, men n (%)	5503 (29.3)***	150888 (25.1)	9695 (42.2)***	288253 (37.8)
Education >12 years, women n (%)	5573 (29.1)***	151362 (24.4)	10790 (46.9)***	329121 (43.2)

Source: Statistics Sweden and Public Health Agency of Sweden

^¤^ Mean income data from 1991 in both Sollentuna Municipality and Stockholm County.

^§^ High income was defined as the aggregate income of the 20 percent highest and low income was defined as the aggregated income of the 20 percent lowest in Sweden. All persons aged 20–64 years of age with an income of zero Swedish crowns and above were included.

*** p<0.001 comparison between Sollentuna Municipality and Stockholm County.

### Data sources

Aggregated outcome data were obtained from the Cause of Death Register, and the Swedish National Hospital Discharge Register, which contains more than 99% of all somatic and psychiatric hospital discharges in Sweden from 1987 and onwards [20]. Data from the National AMI Register were also used [21]. This register contains all subjects with an AMI diagnosis reported to either the Hospital Discharge Register or the Cause of Death Register. The quality of the register has been shown to be very high [20–23]. The characteristics of the population in Sollentuna Municipality and Stockholm County in 1990 and 2010 were obtained from Statistics Sweden (2014) [24] and the Public Health Agency of Sweden [25] (Table 1). Data relating to municipalities in Stockholm were obtained from Statistics Sweden, the Public health agency of Sweden, and Stockholm County Council [24–27].

All-cause and AMI mortality as well as hospitalizations for AMI (ICD-9 code (1987–1996) 410 and ICD-10 code (1997-) I21 and I22), were obtained for the population of Sollentuna and Stockholm, using national registers [28].

### The cardiovascular disease prevention program

In 1988, three health-care centers started an individual and population-oriented cardiovascular prevention program in Sollentuna (N = 50,242, 50.7% women). Between August 8, 1988 and December 10, 1993, persons (n = 5938, 35.7% men) who visited the health-care centers, with or without appointments, regardless of reason for contact, were offered a health check with the emphasis on lifestyle and cardiovascular risk factors. The participants aged between 15 and 86 years, and with an average age of 45.5 years (men 46.2 years and women 45.1 years) answered a questionnaire on life-style and cardiovascular risk factors. Height, weight, waist and hip circumference, blood pressure, fasting blood lipids and blood glucose were measured. Those with one or more cardiovascular risk factor, according to established Swedish guidelines at that time, were offered individual counselling with the emphasis on lifestyle changes by a physician and/or a nurse [19]. The cut-off values were: body mass index (BMI) ≥25 kg/m^2^, smoking, diastolic blood pressure ≥90 mmHg, s-cholesterol ≥5.2 mmol/l, and s-triglycerides ≥2.3 mmol/l. Values of s-cholesterol between 5.2 mmol/l and 6.4 mmol/l were regarded as borderline hypercholesterolemia and these patients received only general advice and no follow-up, while s-cholesterol levels of ≥6.5 mmol/l were regarded as a risk factor which required advice, treatment and follow-up. These patients were also given the opportunity to participate in groups which focused on weight reduction, smoking cessation and physical exercise. Co-operation with a local sports club in Sollentuna was initiated, and physical activity on prescription was started. Within a few years, more than 25 different custom exercise groups had been initiated [16,19]. Furthermore, during a period of 17 years, open lecture series were offered once weekly to patients and their families. Before the start of the project and during the first four years, training was offered to all staff in primary care on repeated occasions. The training included scientific background, treatment according to the current guidelines for CVD prevention and education on how to improve lifestyle and pedagogic skills.

In parallel with the individualized work conducted during the first eight years, comprehensive population-oriented work was undertaken. On about 70 different occasions, key people from grocery stores, nurseries, schools, the municipality, public dental care and different associations in the municipality were invited to educational meetings on how to improve lifestyle and prevent cardiovascular disease. Several collaborative projects were initiated.

### Ethics statement

All data were delivered from registers in aggregated form, and no individual patient consent was required. Within the project it was not possible to provide individual information and obtain individual informed consent from the whole study population of Stockholm County. The Regional Ethical Review Board in Stockholm approved the project (reference number: 2012/1172-31/1 and 2013/1329-32).

The study is registered at www.clincaltrials.gov. The authors confirm that all ongoing and related studies are registered.

### Statistical analyses

Incidence and mortality rates were calculated by calendar year, gender and age, using the mean population as the denominator. The data were divided into five calender-year groups (1987–1989, 1990–1994, 1995–1999, 2000–2004, 2005–2010). Rates were expressed per 100,000 person years and were age standardized using direct standardization andthe population of Sweden in 2000 as standard population. To test for differences in trend, an interaction term between groups (Sollentuna vs Stockholm) and time was included. All effects were evaluated with the F-test. No tests for competing risk were taken into consideration.

In presentation of the background data differences between Sollentuna and Stockholm were tested using chi-square tests (categorical variables) and Student´s t-test (continuous variables).

The observed numbers of prevented or postponed deaths were calculated by subtracting the number of all-cause deaths per 100,000 people in Sollentuna and Stockholm in 1987–1989 from those in 2005–2010. Statistical analyses were performed using SPSS version 19.

## Results

During the observation period, the numbers of AMI cases registered were 3,207 (60% men) in Sollentuna, and 135,958 (58% men) in Stockholm. AMI deaths occurred in 1,011 persons (58% men) living in Sollentuna and 50,365 persons (55% men) living in Stockholm. All-cause death was registered in 8,504 persons (50% men) in Sollentuna and 370,295 persons (48% men) in Stockholm.

**Fig 1:**
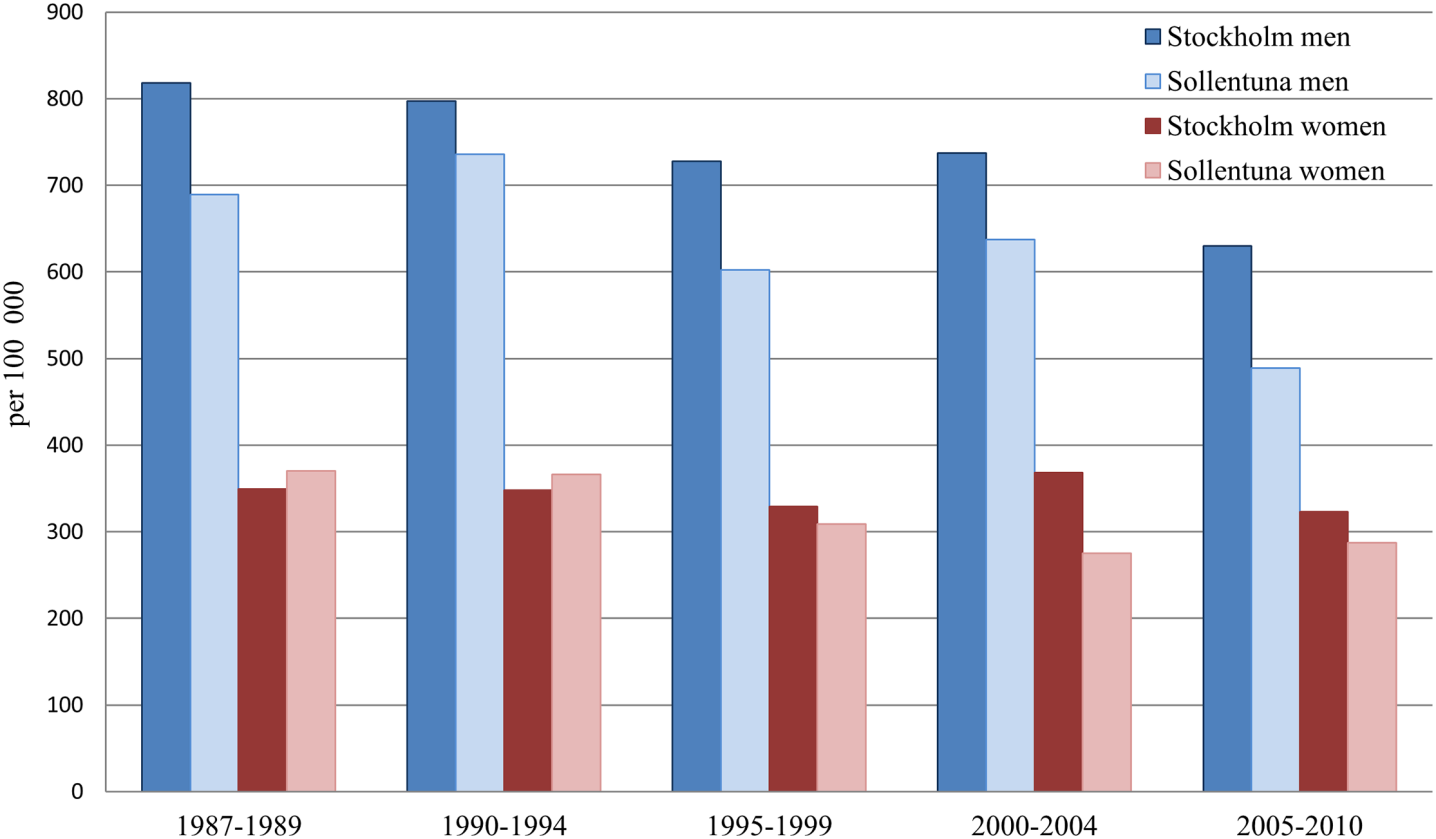
Age standardized AMI incidence in men and women per 100 000 inhabitants in Stockholm and Sollentuna in five year groups between 1987 to 2010.

**Fig 2:**
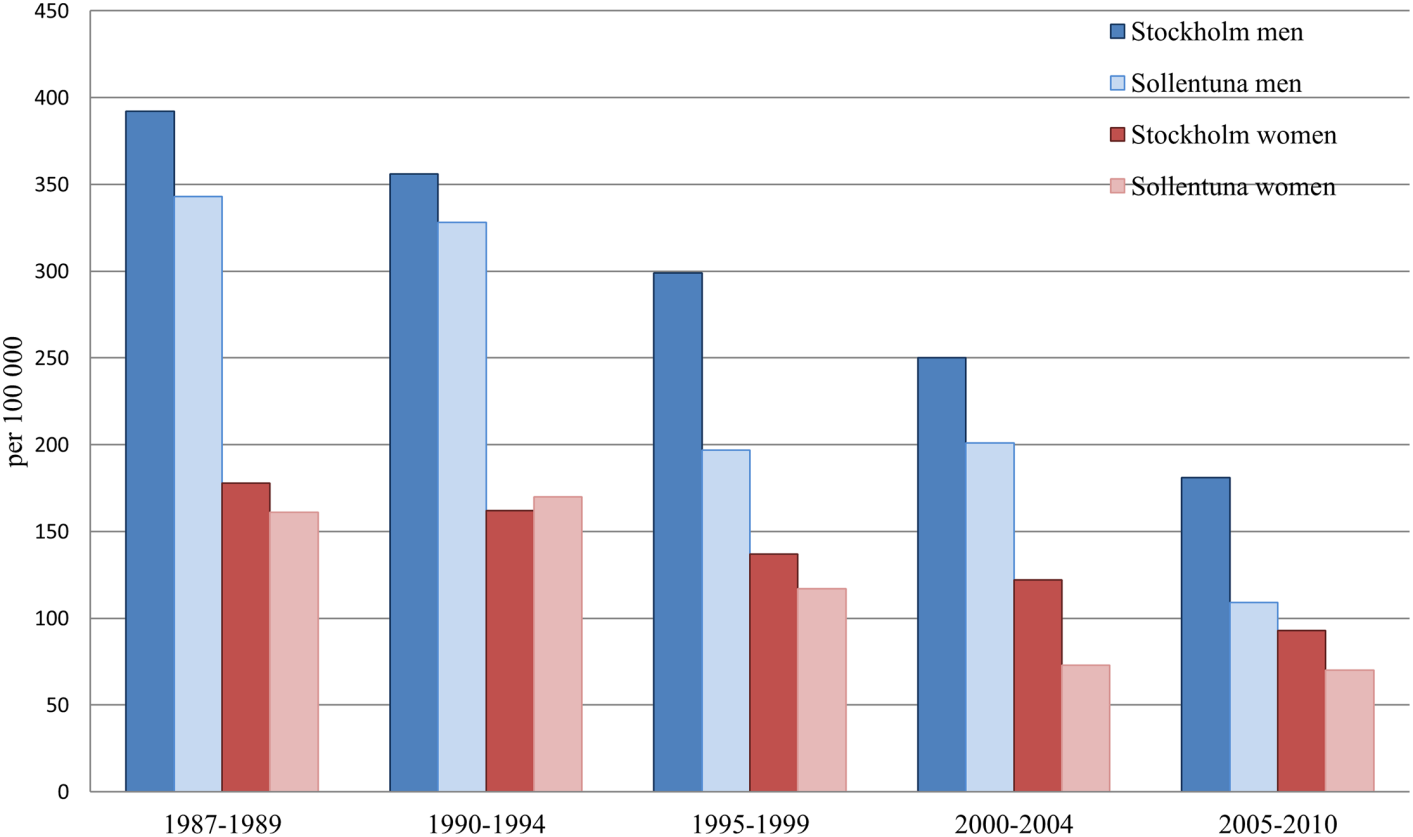
Age standardized AMI mortality in men and women per 100 000 inhabitants in Stockholm and Sollentuna in five year groups between 1987 to 2010.

**Fig 3:**
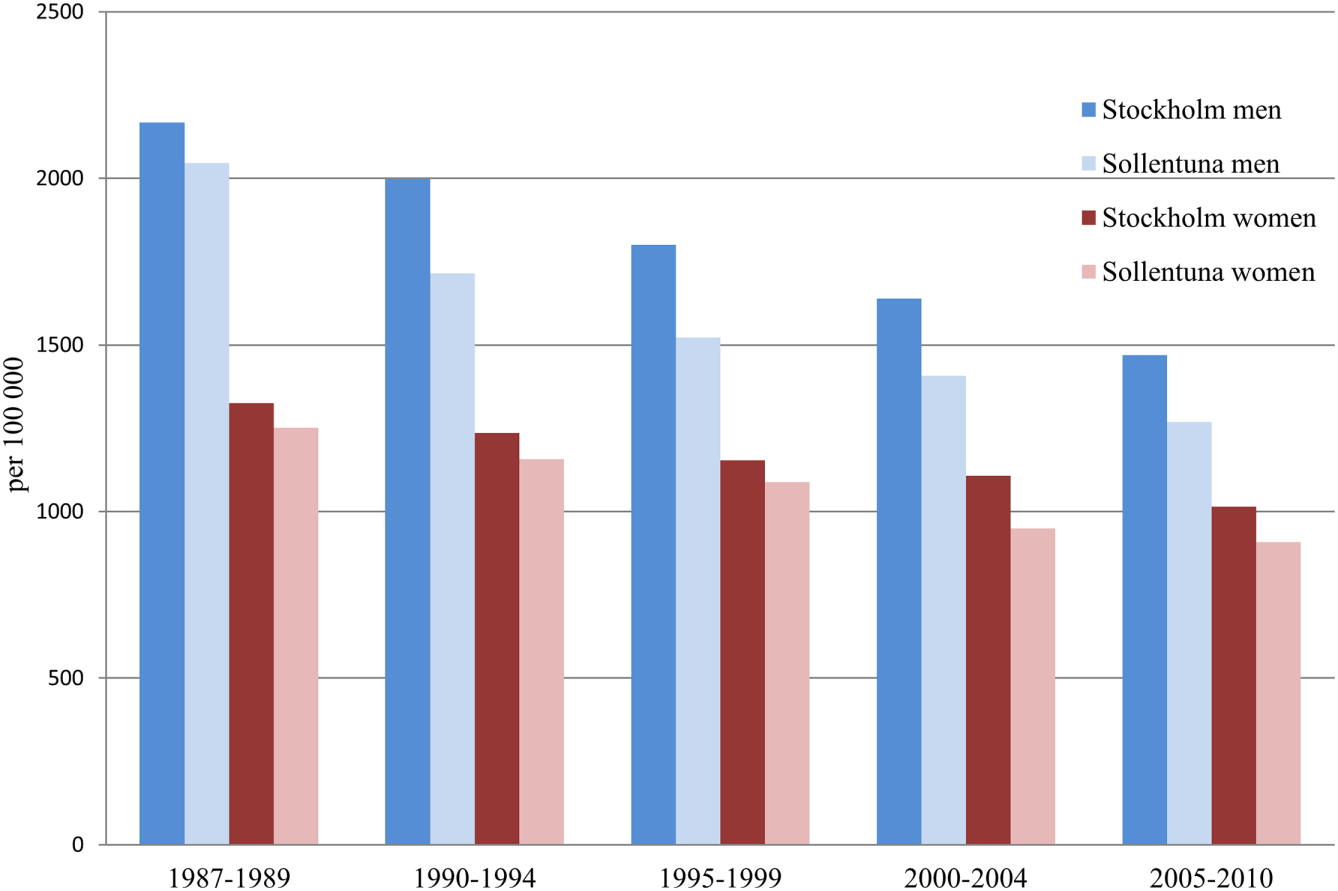
Age standardized all-cause mortality in men and women per 100 000 inhabitants in Stockholm and Sollentuna in five year groups between 1987 to 2010.

**Fig 4:**
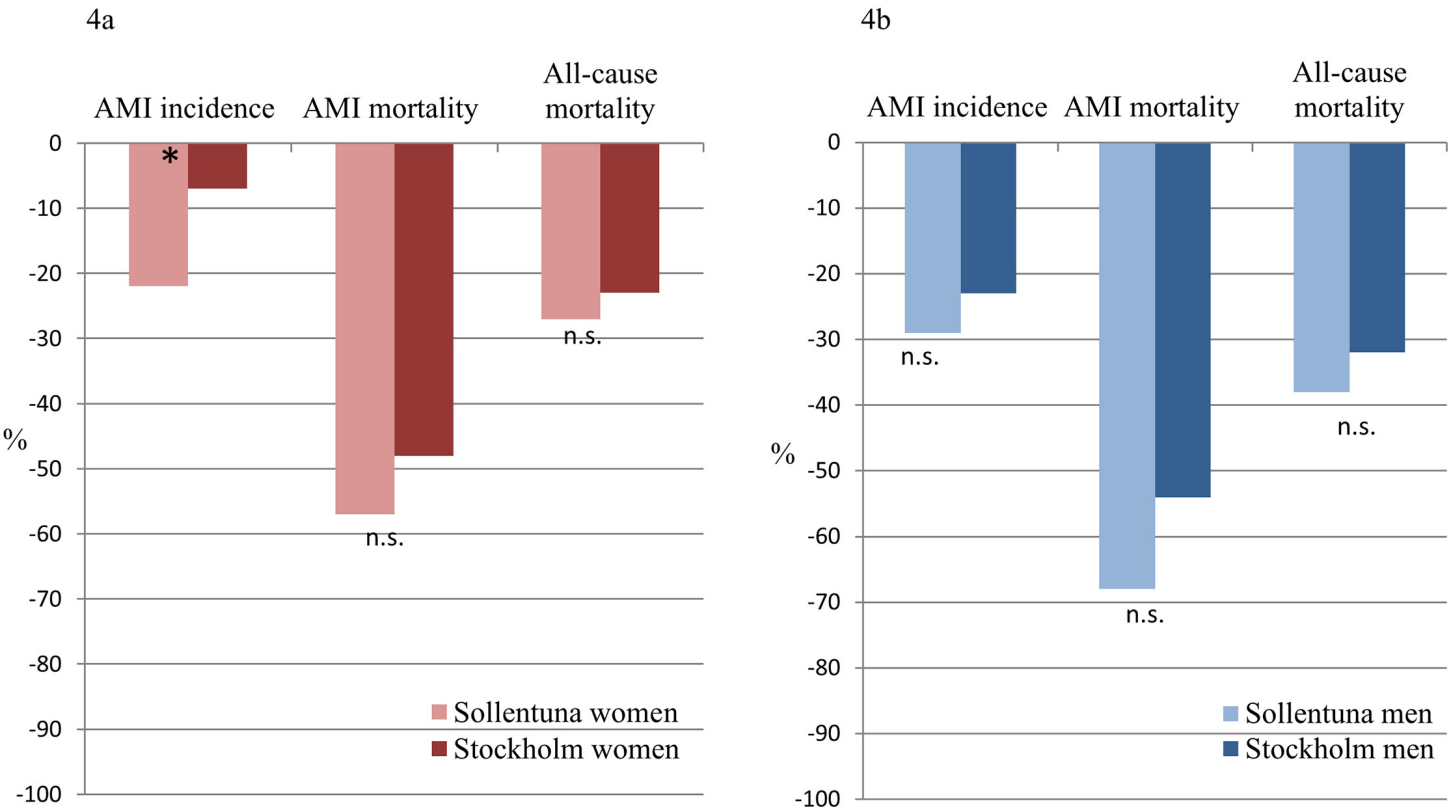
Title. Relative changes (%) of AMI incidence, AMI mortality, and all-cause mortality in women (Fig 4a) and men (Fig 4b) in Sollentuna and Stockholm between 1987–1989 and 2005–2010. Age standardized rate. AMI; acute myocardial infarction, n.s.; not significant, * p<0.05.

The AMI incidence, AMI mortality, and all-cause mortality declined substantially in Sollentuna as well as in Stockholm during the observation period (Figs 1–3). In women from Sollentuna, the AMI incidence declined significantly more compared with women in Stockholm (-22% vs -7%, difference in slope p<0.05) (Fig 4a). There was also a greater decline in women from Sollentuna compared to Stockholm in AMI mortality (-57% vs. -48%), and all-cause mortality (-27% vs. -23%) but the differences were not significant. In men from Sollentuna compared to men from Stockholm there was a greater decline in AMI incidence (-29 vs. -23%), AMI mortality (-68 vs. -54%) and all-cause mortality (-38% vs. -32%) but the differences were not significant (Fig 4b).

Between 1987–1989 and 2005–2010, the AMI mortality decreased in Sollentuna men (from 343 to 109 per 100,000), in Stockholm men from 392 to 181 per 100,000, in Sollentuna women from 161 to 70 per 100,000 and in Stockholm women from 178 to 93 per 100,000. The all-cause mortality rate decreased more in both men and women from Sollentuna men from 2,046 to 1,268, and women 1,251 to 909 per 100,000 compared with Stockholm men from 2,168 to 1,469, and women 1,325 to 1,015 per 100,000. In Sollentuna compared with Stockholm, 79 fewer men and 32 fewer women per 100,000 died between 1987–1989 and 2005–2010.

The decline in all-cause mortality between 1987–1989 and 2005–2010, resulted in a large number of prevented or postponed deaths in Sollentuna and Stockholm respectively (n = 1,120 vs. 1,009 per 100,000 persons).

## Discussion

This study covering more than 20 years follow-up shows that the incidence of AMI decreased more in women living in Sollentuna compared with women living in Stockholm in 1987–2010. The AMI incidence in men, as well as AMI and all-cause mortality for both men and women, declined and there were trends towards a greater decline (n.s.) in Sollentuna Municipality compared with the rest of Stockholm County.

The average life expectancy has increased continuously during the last few decades in Sweden. In 2011, the life expectancy was 79.8 years for men and 83.7 years for women in Sweden [28]. The main reason for this was the reduction in CVD mortality, which was more pronounced in men than in women [28]. More than half of the decline in all-cause mortality between 1986 and 2002 in Sweden was due to the decline in CHD mortality, which has decreased by around two-thirds in Sweden over the last two decades in both men and women [8]. Reductions in major risk factors such as smoking, blood pressure, and especially cholesterol have contributed to this [10]. The improved medical treatment and care of people with acute myocardial infarction is thought to explain one third of the decrease in CHD-mortality [10]. Between 1990 and 2010, daily smoking declined from 25% in men and 26% in women to 13% in both sexes in Stockholm County [27]. A previously reported follow-up of the CVD prevention program in Sollentuna showed that men and women with increased levels of blood pressure and blood lipids at entry had a significant reduction in diastolic blood pressure (men -5%, women -6%), cholesterol (men -7%, women -10%) and triglycerides (men -24%, women -42%) [16]. Women had a larger reduction in these three risk factors, which indicated that women appeared to be more motivated than men to follow the prevention program, which is also consistent with the observed reduced risk of AMI. More women than men smoked at baseline (men 28%, women 30%), but unfortunately since there was a large amont of missing data at the follow-up, it was not possible to assess the effect of the program on smoking [16].

Few studies which have evaluated the long-term effects of CVD prevention programs have been published. Studies from the USA (Stanford Five Project and Minnesota Heart Health Program) failed to show any differences in trends for CVD morbidity and mortality between the intervention and control group [29, 30]. However, the thirty-five-year follow-up of an intervention study in North Karelia in Finland showed a larger decline in CHD compared with Finnish people in general, which was associated with reductions in common CVD risk factors [31–33]. The large decline in CHD in Iceland between 1981 and 2006 was also explained by a decrease in major risk factors [7].

Almost all the risk factors for MI, such as hypercholesterolemia, hypertension, type 2 diabetes mellitus, and abdominal obesity, as well as an unhealthy diet, low physical activity, high alcohol consumption, smoking and psychosocial factors can be modified [34, 35]. Guidelines on CVD prevention are now focusing more heavily on the importance of lifestyle counseling and multidisciplinary programs to lower the risk of CVD [36, 37].

### Limitations

Misclassification of CHD deaths may occur in particular among deaths occuring outside hospital [38]. However, the autopsy rate for fatal events outside hospital is high (80%) during the observation period of this study. More sensitive diagnostic criteria for AMI were introduced in health care in Sweden in 2001 and this slightly increased the observed incidence [20]. In 2003, the level was about eight per cent above the level for 2000. However, in 2004, the age-standardized incidence rate for both men and women was reduced by about six per cent and the declining trend continued during the study period for both the incidence ofmortality from AMI [21]. One possible contributing explanation for the somewhat more pronounced decrease in AMI mortality could be that the population in Sollentuna had to a greater extent a high socioeconomic status compared with Stockholm County in general.

In Western Europe, including Sweden the data have shown a more rapid decline of CVD in mortality in higher socioeconomic groups [39]. Low compared with high educational level have been shown to increase the risk of suffering a myocardial infarction [39]. We were not able to make adjustments for education in our aggregated dataset.

We studied six municipalities (including Sollentuna) in Stockholm County with the largest proportions of people with a high education level in 1990 and used education as a socioeconomic indicator. The increase in the proportion of men and women with high education level between 1990 and 2010 was similar to Sollentuna or higher in the other municipalities (S1 and S2 Figs). The decreases in age-standardized AMI incidence (S3 and S4 Figs), and AMI mortality (S5 and S6 Figs) were somewhat more pronounced in both men and women from Sollentuna than in the other municipalities with a comparatively high proportion of the population with high education between 1990–1994 and 2005–2010. The decrease in all-cause mortality was larger for men (S7 Fig) in Sollentuna but not to the same extent for women compared with the other five high educated municipalities (S8 Fig).

Other small-scale local prevention initiatives have been ongoing in Stockholm County, and this may have influenced the result [40].

The decline in AMI and all-cause mortality was fairly similar in both men and women from Sollentuna and Stockholm during the study period. However, consistent trends towards a larger decline, especially in Sollentuna, were found in all the studied outcomes for both genders. There were fairly large differences in the decline in the incidence of AMI, AMI mortality, and all-cause mortality between the municipalities in Stockholm County [25]. It is possible that the prevention program influenced not only Sollentuna Municipality but also the surrounding area.

The strengths of our study were that the entire populations of the county and municipality were included and that registries of hospital discharges and deaths had a high level of completeness.

## Conclusion

The decline in AMI incidence and mortality and all-cause mortality was pronounced, in both men and women from Sollentuna and the rest of Stockholm, during this long-term follow-up of a cardiovascular prevention program. The AMI incidence declined significantly more in women from Sollentuna, which may indicate a positive effect of the individual and population-oriented cardiovascular prevention program.

## Supporting Information

S1 Fig Proportion of men, with high education (>12 years) in 1990 and 2010 in six municipalities and Stockholm County. (TIF)

S2 Fig Proportion of women, with high education (>12 years) in 1990 and 2010 in six municipalities and Stockholm County. (TIF)

S3 Fig AMI incidence in men in six municipalities and Stockholm County. (TIF)

S4 Fig AMI incidence in women in six municipalities and Stockholm County. (TIF)

S5 Fig AMI mortality in men in six municipalities and Stockholm County. (TIF)

S6 Fig AMI mortality in women in six municipalities and Stockholm County. (TIF)

S7 Fig All-cause mortality in men in six municipalities and Stockholm County. (TIF)

S8 Fig All-cause mortality in women, in six municipalities and Stockholm County. (TIF)

S9 Fig Proportion (%) of persons, with high education (>12 years) in 26 municipalities of Stockholm County. (TIF)

S10 Fig Flow chart of study population. (TIF)

S1 Text Check list trend statement. (PDF)

S2 Text The study protocol in Swedish. (PDF)

S3 Text The study protocol in English. (PDF)
