# Supplementary figures and images for: Time Trends in Incidence and Mortality of Acute Myocardial Infarction, and All-Cause Mortality following a Cardiovascular Prevention Program in Sweden
Source: PLoS One. 2015 Nov 18;10(11):e0140201. doi: 10.1371/journal.pone.0140201 (PMC4651336; doi:10.1371/journal.pone.0140201)

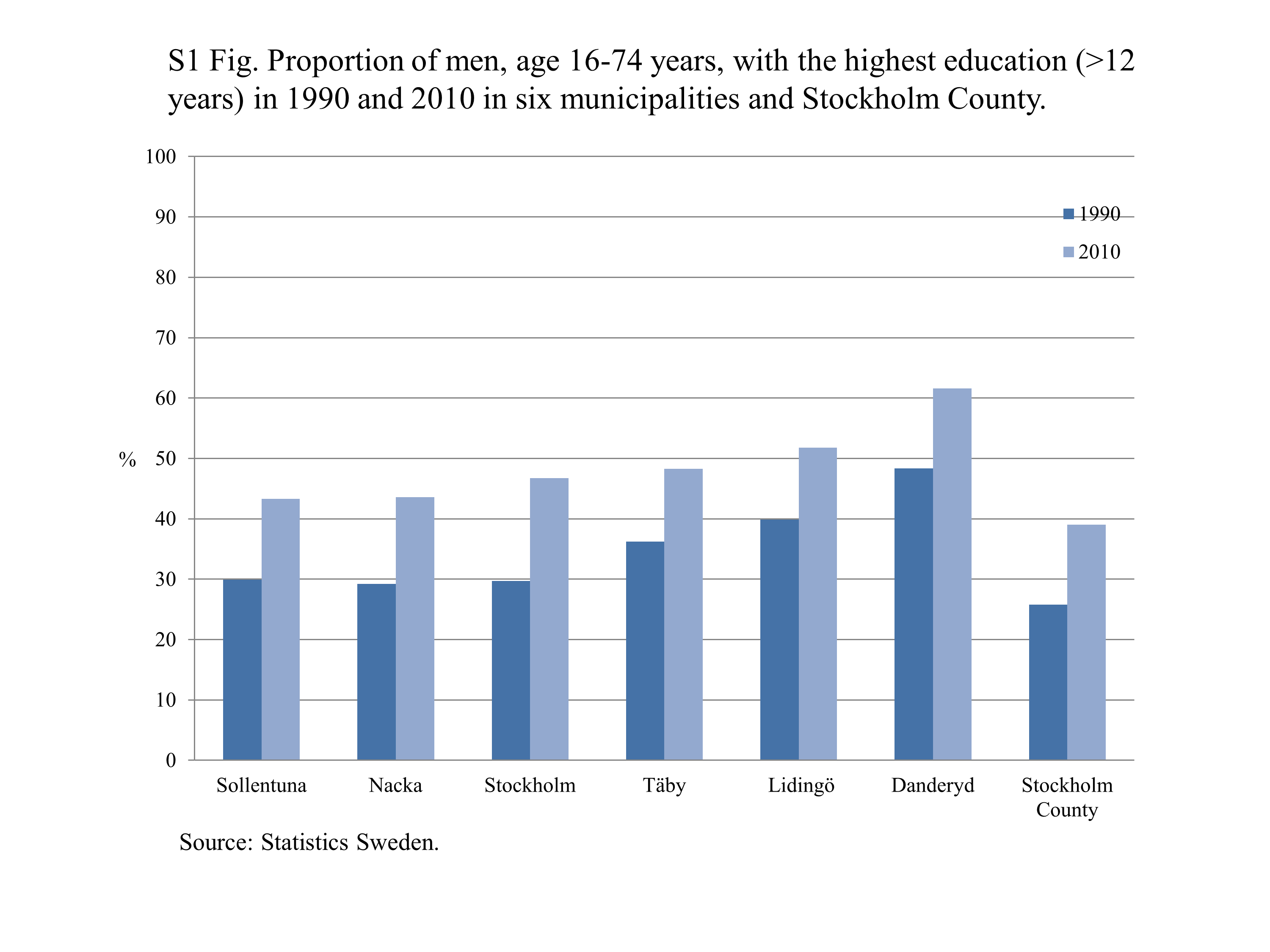

Supplement: S1 Fig — (TIF) [file pone.0140201.s001.tif]

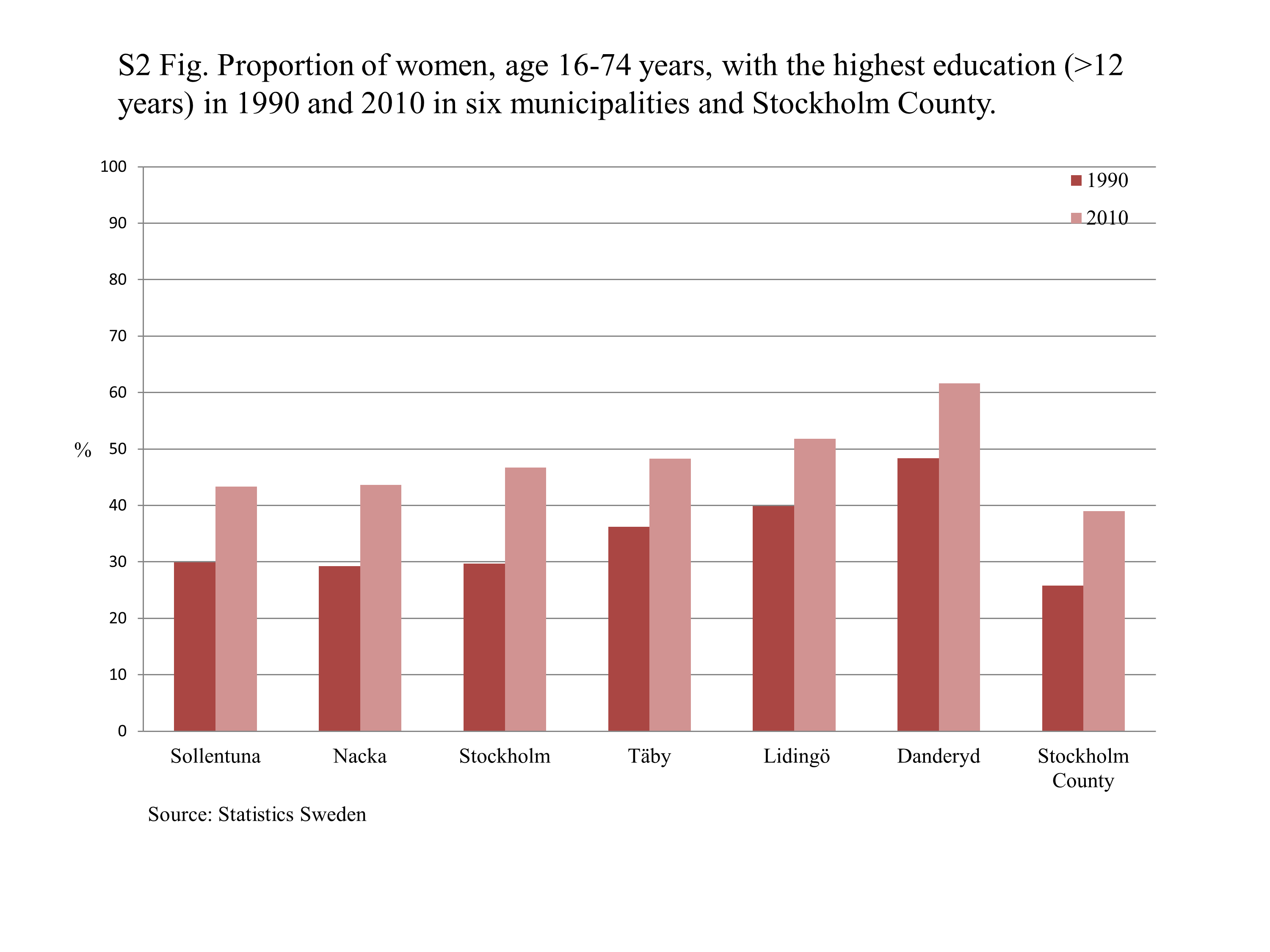

Supplement: S2 Fig — (TIF) [file pone.0140201.s002.tif]

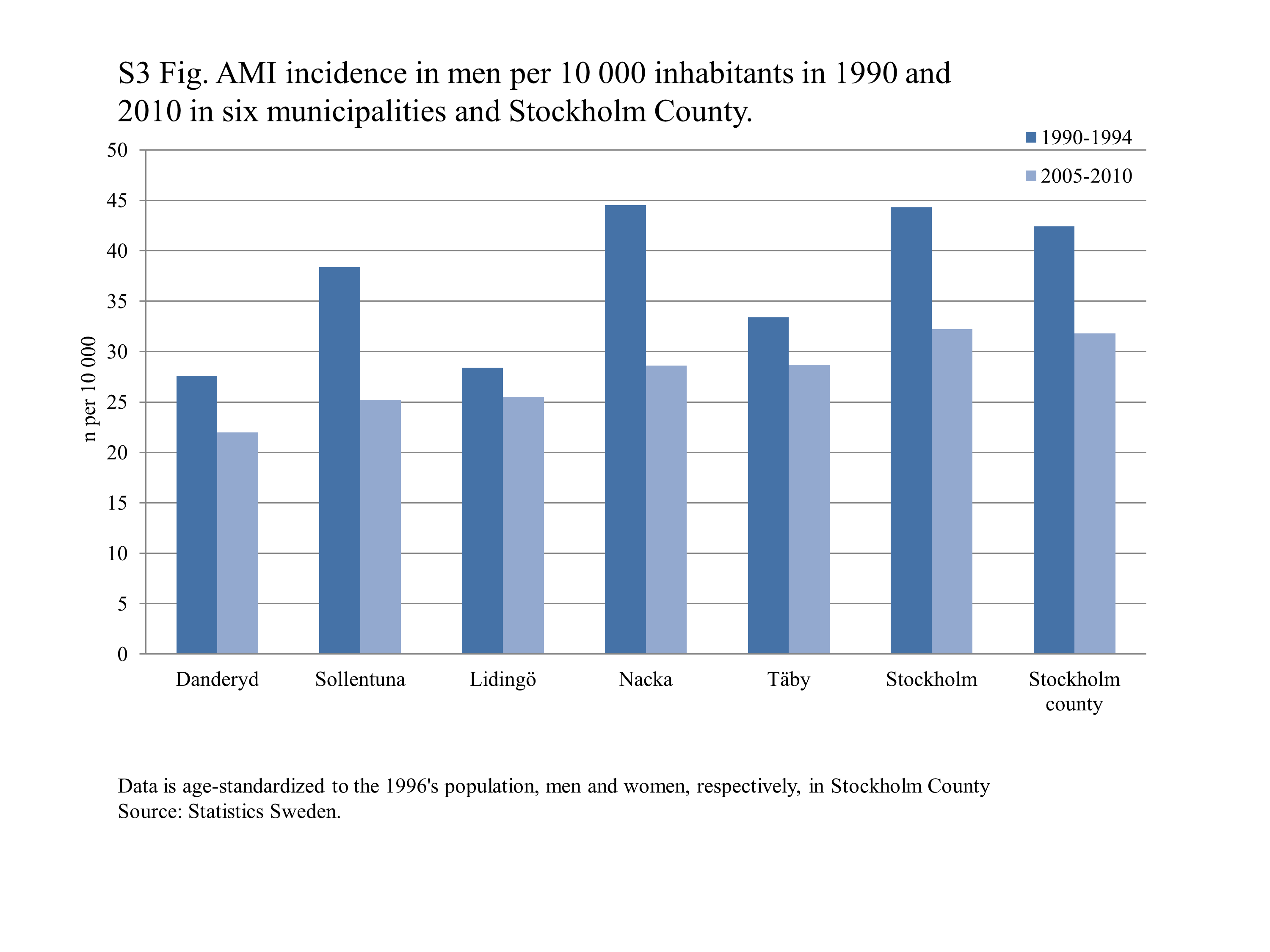

Supplement: S3 Fig — (TIF) [file pone.0140201.s003.tif]

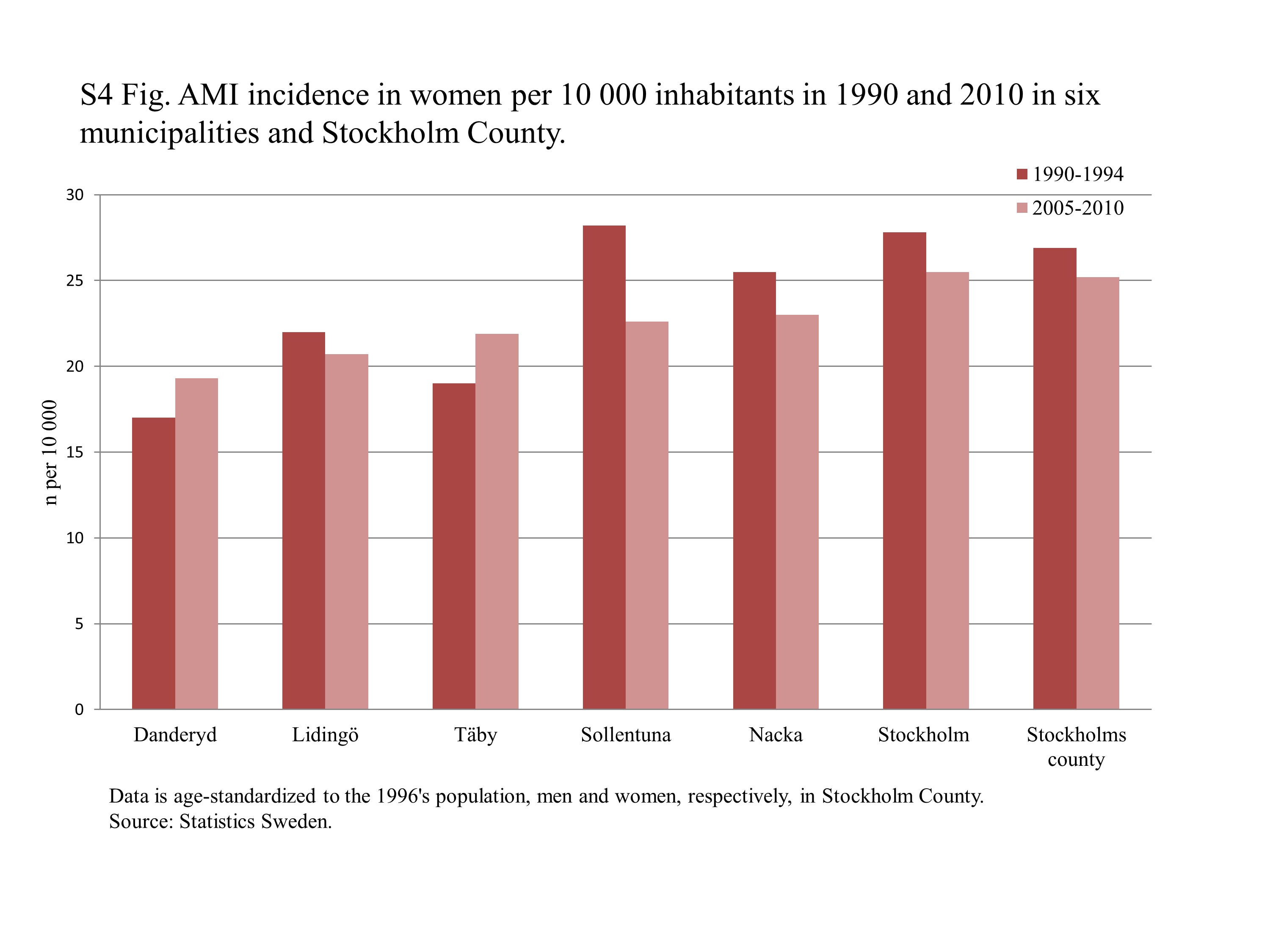

Supplement: S4 Fig — (TIF) [file pone.0140201.s004.tif]

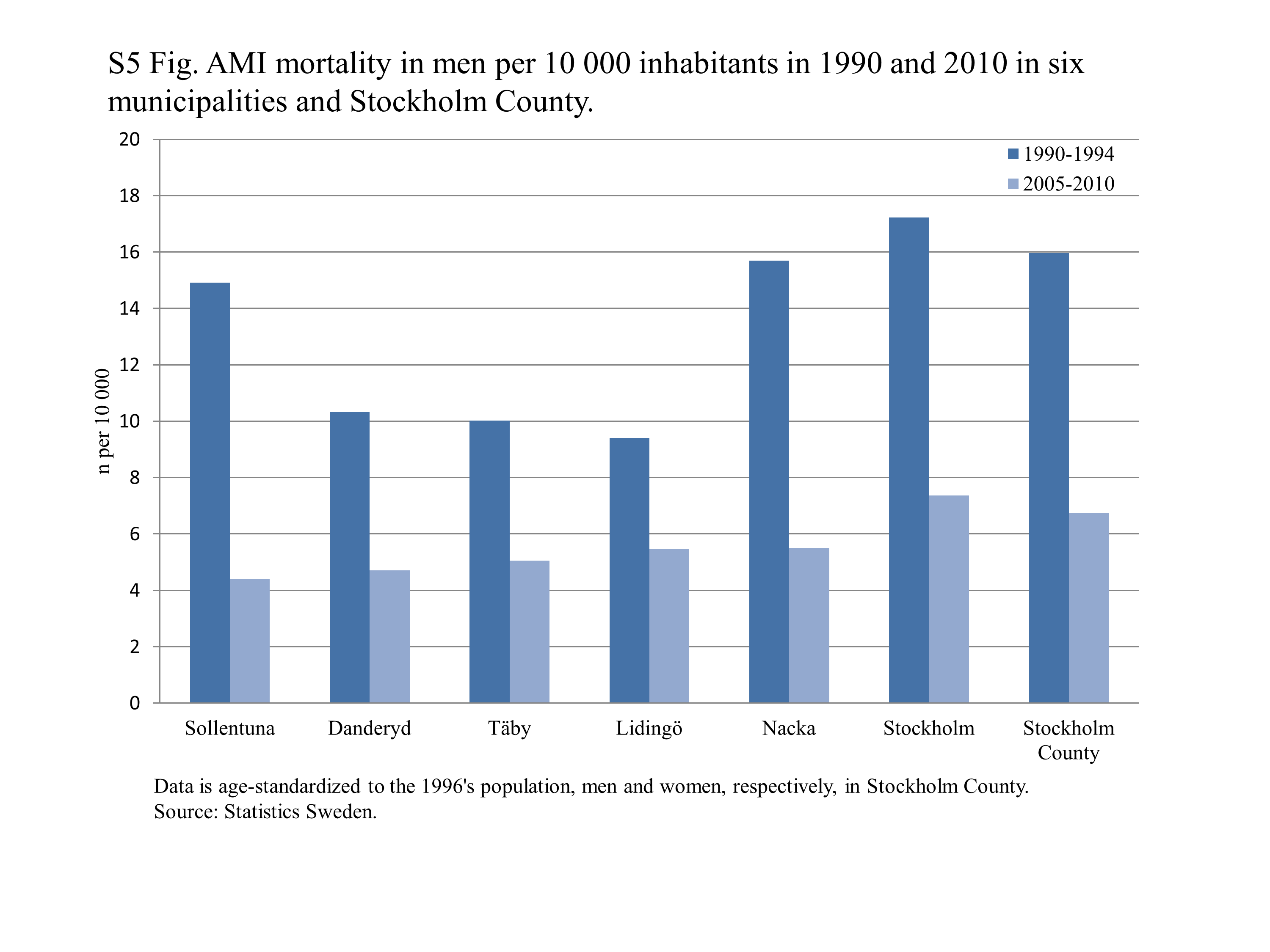

Supplement: S5 Fig — (TIF) [file pone.0140201.s005.tif]

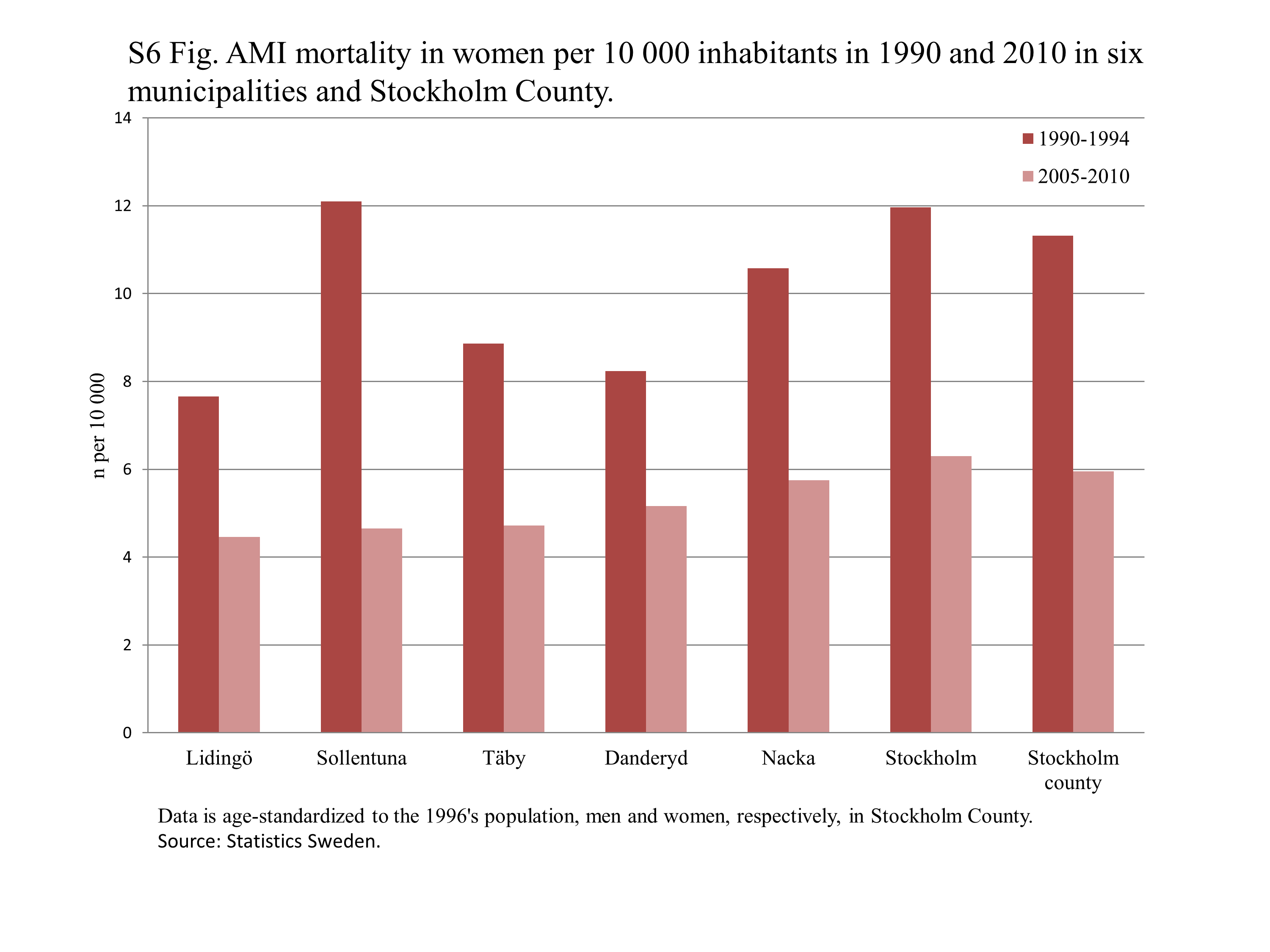

Supplement: S6 Fig — (TIF) [file pone.0140201.s006.tif]

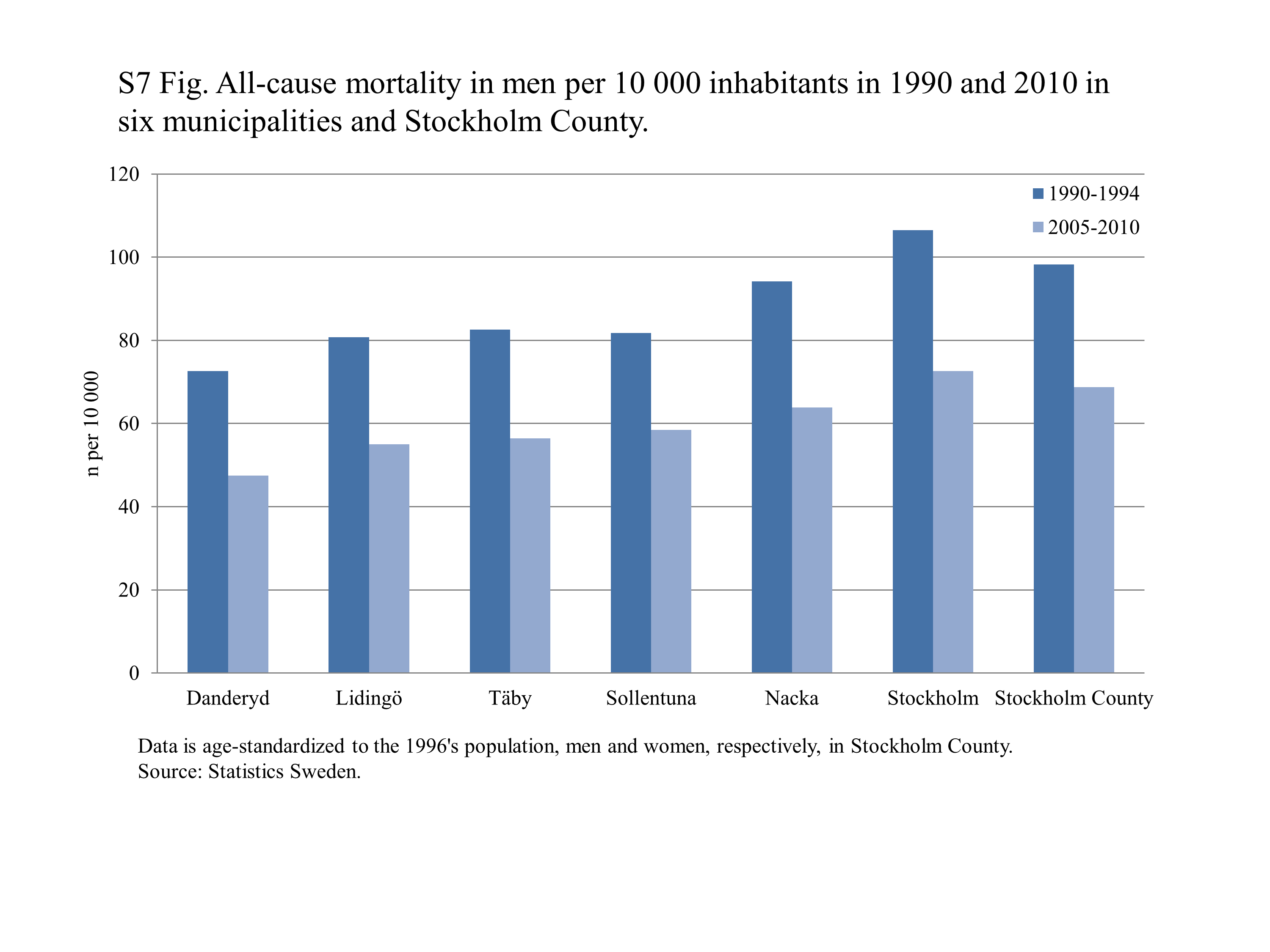

Supplement: S7 Fig — (TIF) [file pone.0140201.s007.tif]

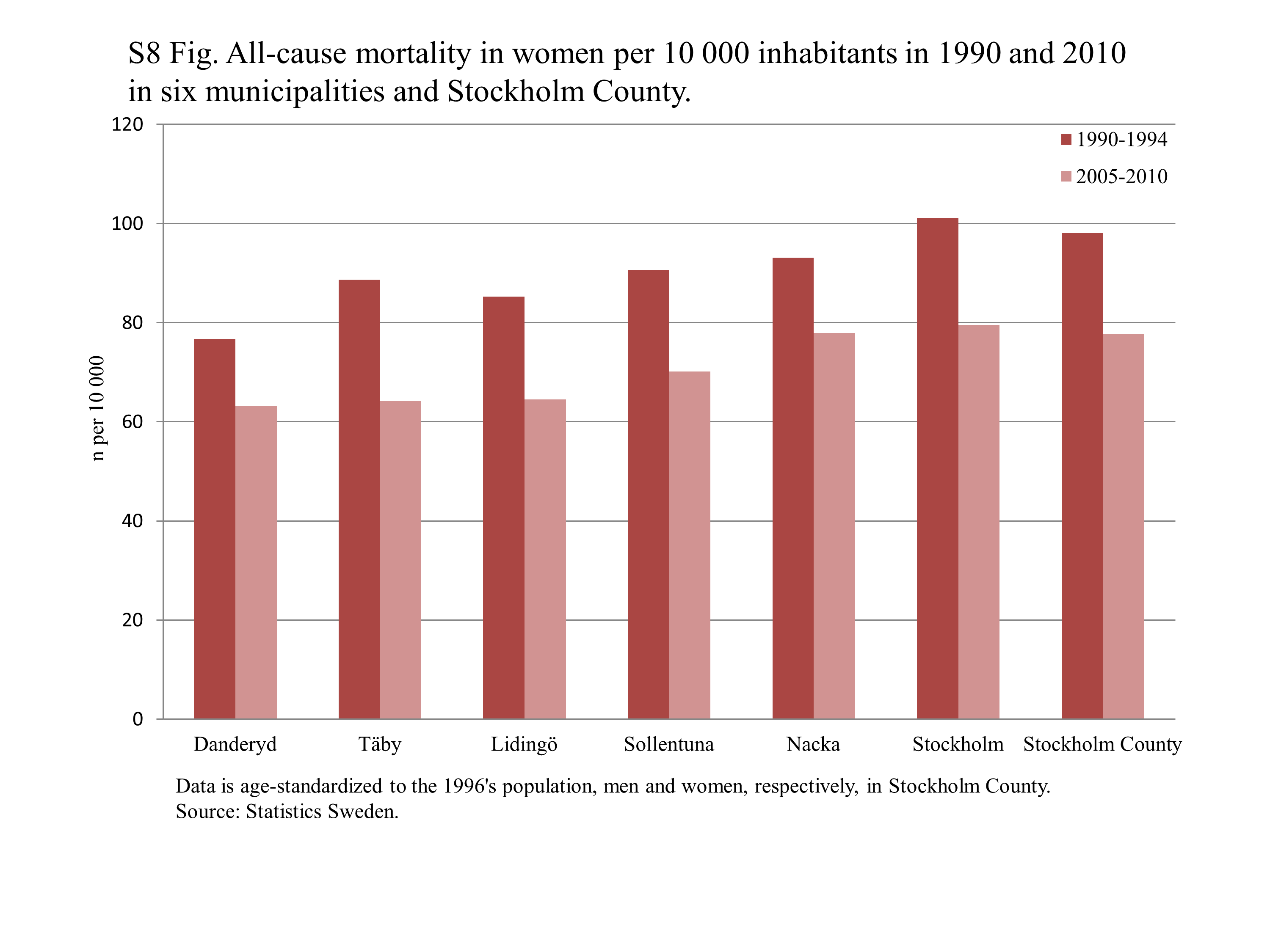

Supplement: S8 Fig — (TIF) [file pone.0140201.s008.tif]

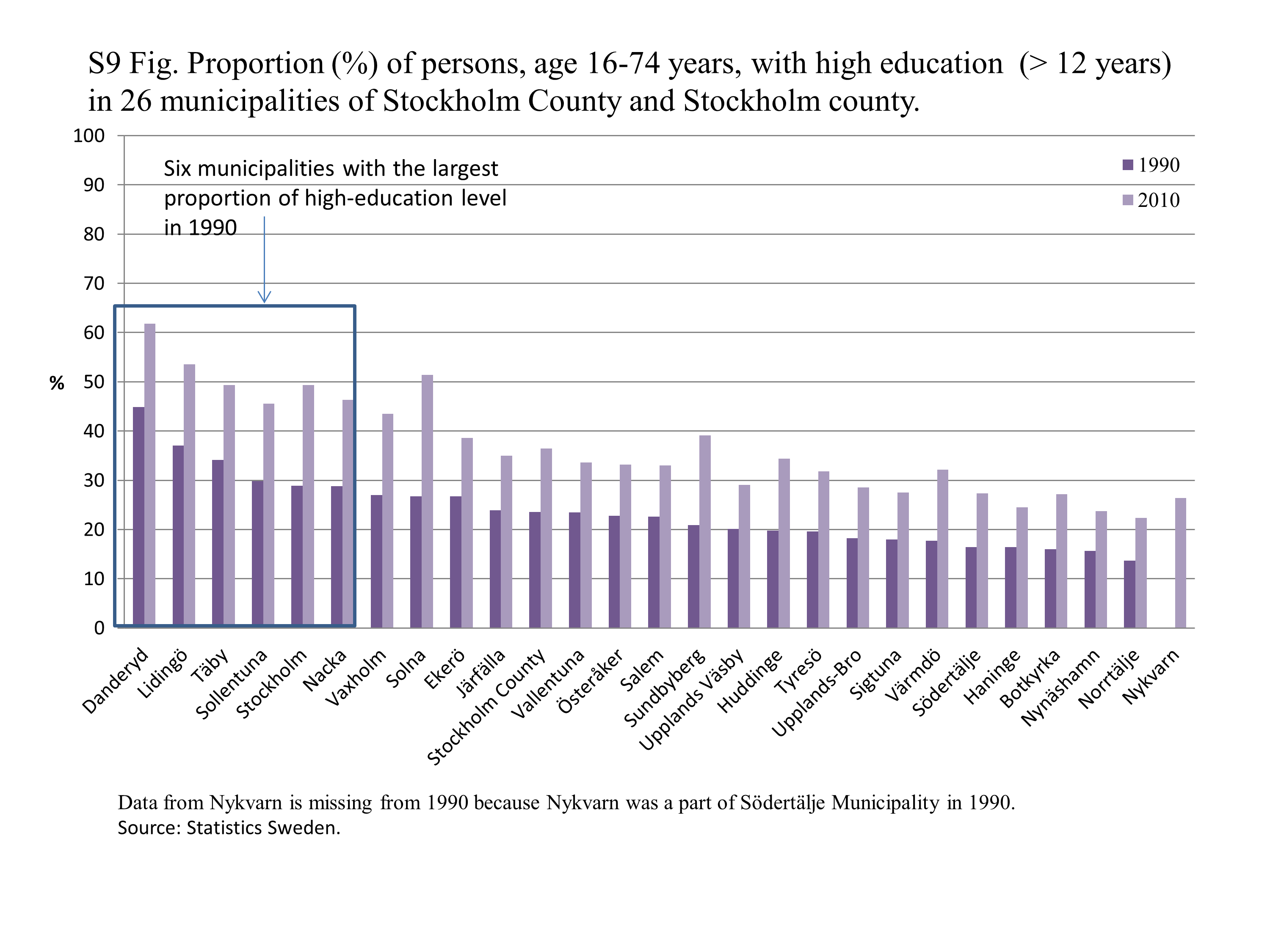

Supplement: S9 Fig — (TIF) [file pone.0140201.s009.tif]

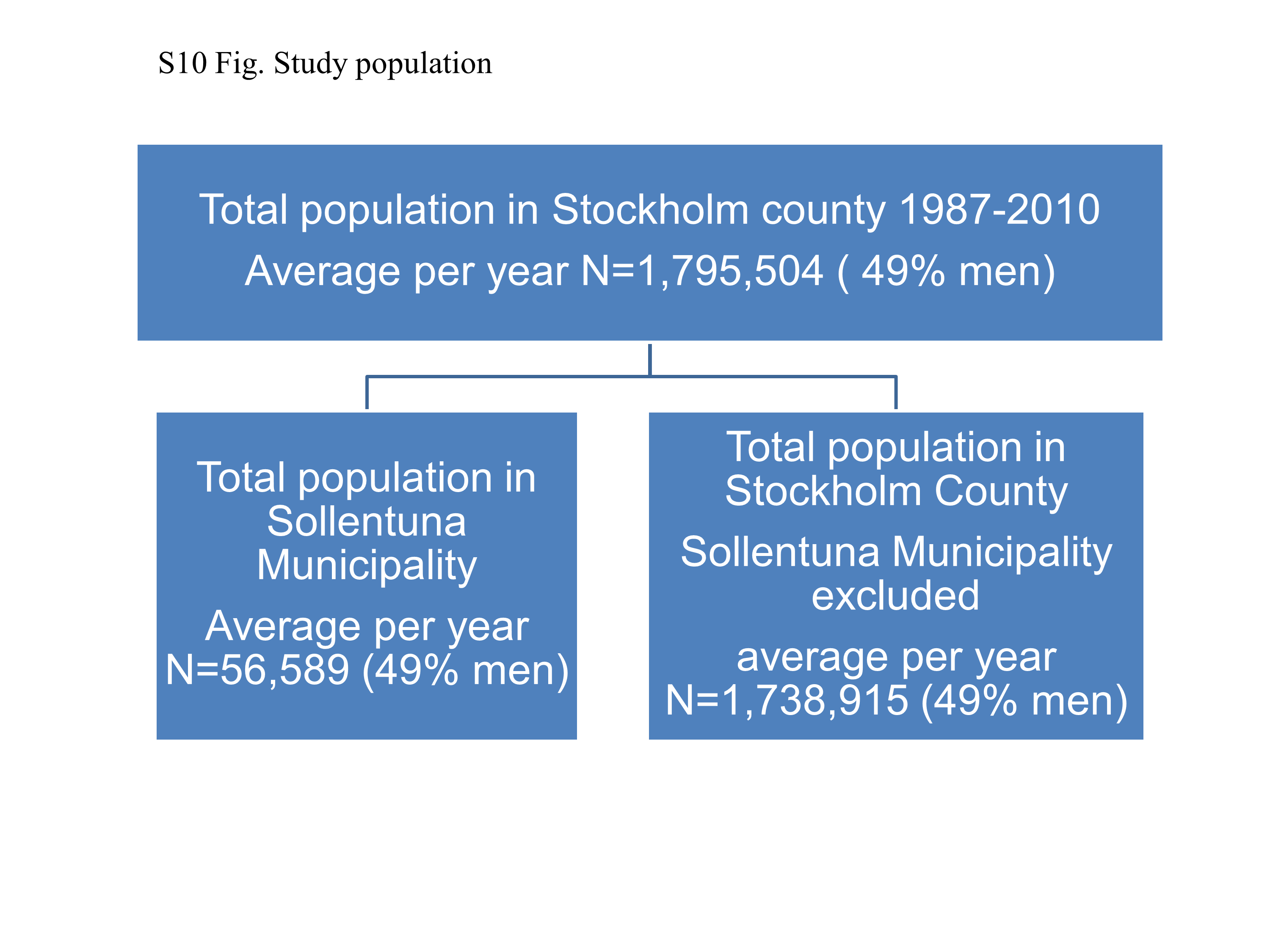

Supplement: S10 Fig — (TIF) [file pone.0140201.s010.tif]
