# Supplementary material for: Time Trends in Incidence and Mortality of Acute Myocardial Infarction, and All-Cause Mortality following a Cardiovascular Prevention Program in Sweden
Source: PLoS One. 2015 Nov 18;10(11):e0140201. doi: 10.1371/journal.pone.0140201 (PMC4651336; doi:10.1371/journal.pone.0140201)
Supplement: S2 Text — (PDF) [file pone.0140201.s012.pdf]

Projektplan

Uppföljning av Sollentuna prevention program (SoPP)

Gunilla Journath [1], Niklas Hammar [2,3], Ulf de Faire [2], Peter Lindgren[2] , Ingvar Krakau[1], Stig Elofsson [4], Göran Walldius[2] och Mai-Lis Hellénius [1].

1. Institutionen för medicin, Karolinska Institutet, Karolinska Universitetssjukhuset, 17176 Solna
2. Institutet för Miljömedicin, Nobels väg 13, Karolinska, Institutet, Solna, 171 77 Stockholm
3. Patient Safety, Epidemiology, AstraZeneca R & D, Södertälje and Mölndal, Sweden
4. Stockholm Universitet, Universitetsvägen 10A, 106 91 Stockholm

Bakgrund

Trots att incidens och mortalitet av kardiovaskulära sjukdomar sjunkit under de senaste decennierna drabbades och avled 41 procent av kvinnorna och 39 procent av männen under 2010 i Sverige av dessa sjukdomar (1,2).

De bakomliggande faktorerna är till stor del kända (3-5). Förebyggande insatser med fokus på livsstilsförändring har fått en allt mer central plats i hälso- och sjukvården. Incidens och mortalitet har sjunkit. Behandling inom vården har beräknats svara för 30-40% och livsstilsförändringar för minst 50% (6). Därför fokuserar riktlinjer allt mer på livsstilens betydelse (7). Storskaliga preventionsprogram har genomförts i svensk primärvård, t.ex. i Västerbotten, Habo och Sollentuna (8-10). Dock är utvärderingar av långsiktiga effekter på kardiovaskulär hälsa, liksom hälsoekonomiska utvärderingar, fortfarande få.

Fyra vårdcentraler, i Sollentuna kommun norr om Stockholm, erbjöd besökare, oavsett kontaktorsak, en hälsokontroll med fokus på livsstil och kardiovaskulära riskfaktorer under perioden 1988-92. Ett enkelt frågeformulär fylldes i (bil1) (11). Längd, vikt, midje- och stussmått, blodtryck samt blodfetter (fastande) kontrollerades. Hos riskindivider togs även fasteblodsocker (bil 2). Individer med förhöjda nivåer av nämnda riskfaktorer erbjöds individuell rådgivning med fokus på livsstilsförändring hos läkare och/eller sköterska (bil.3). Möjlighet att delta i överviktsgrupper, rökavvänjningsgrupper och anpassad

motionsverksamhet fanns. Ett nära samarbete med Korpen Sollentuna inleddes och inom några år kunde över 25 olika anpassade motionsgrupper varje termin erbjudas patienter som fått remiss till fysisk aktivitet av primärvårdspersonal (föregångare till FaR, fysisk aktivitet på recept). En kväll i veckan erbjöds under 17 år en öppen föreläsningsserie för patienter och anhöriga (10-12).

Före programstart och under de första 4 åren arrangerades utbildning för all personal i primärvården vid ett 40-tal olika tillfällen. Utbildningen innehöll vetenskaplig grund samt pedagogik vid livsstilsförändring (1,3).

Parallellt med det individinriktade arbetet bedrevs under de första 8 åren ett omfattande populationsinriktat arbete. Vid ett 70-tal olika tillfällen inbjöds nyckelpersoner från livsmedelsbutiker, daghem, skolor, kommunen, folktandvården, föreningsliv etc till informationsträffar och undervisning om livsstil och hjärtkärlsjukdom och flera samarbetsprojekt inleddes (10-12).

Sjunkande riskfaktornivåer hos deltagare i preventionsprogrammet

Under det första verksamhetsåret registrerades 2116 individer i programmet (10) och en majoritet (70 %) hade riskfaktorer som krävde behandling och uppföljning. En attitydundersökning bland deltagare visade en positiv attityd till preventionsprogrammet (12). Vid en senare uppföljning av 5622 personer som registrerades i programmet under de första fyra åren (1988-1992) visade sig en hög andel ha riskfaktorer för hjärtkärlsjukdom. Det visade sig att de som initialt hade höga blodfetter eller högt blodtryck signifikant sänkte sina nivåer under en uppföljning på i medeltal 15 månader (13). Blodtrycket sjönk med ca 5 mm Hg hos både män och kvinnor och kolesterolnivåerna med i snitt 7 % hos männen och 10 % hos kvinnorna. En uttalad sänkning av triglycerider motsvarande 24 % hos männen och 42 % hos kvinnorna observerades (13). Framgångsrik sänkning av blodfetter var kopplat till kvinnligt kön och längre utbildning (14).

Effekter av kost och motion på kardiovaskulär risk

Deltagare i preventionsprogrammet har utgjort rekryteringsbas till randomiserade kontrollerade studier där effekter av livsstilsintervention (fr.a. råd om kost och/eller motion)

på kardiovaskulära riskfaktorer och livskvalitet studerats. Ett antal publikationer och tre avhandlingar har framlagts (A. Asplund-Carlsson 1994, M-L Hellénius 1995, GK. Näslund 1996). Lågintensiv intervention rörande matvanor och/eller fysisk aktivitet riktade mot friska medelålders män med förhöjd kardiovaskulär risk hade effekt på vikt, bukfetma, blodtryck, lipider och insulinkänslighet efter sex månader (15,16). Flera av effekterna kvarstod vid en 18-månaders uppföljning (17). Inflammatoriska markörer och adhesionsmolekyler påverkades likaså (Sjögren et al 2011). Vilka faktorer som påverkar benägenheten att delta i kost och motionsprogram, liksom följsamhet till en given intervention har också studerats.

Övergripande målsättning

Att utvärdera långtidseffekter av ett individ- och befolkningsinriktat kardiovaskulärt preventionsprogram i primärvården.

Specifika målsättningar

Att studera om ett individ- och befolkningsinriktat kardiovaskulärt preventionsprogram vid en 20-årig uppföljning hos de intervenerade, deras anhöriga och kommunens befolkning har påverkat

A) incidens och mortalitet i akut hjärtinfarkt, stroke, kardiovaskulär sjukdom (hjärtinfarkt, angina, stroke, hjärtsvikt och/eller perifer kärlsjukdom), samt totalmortalitet.

B) läkemedelsbehandling av diabetes, höga blodfetter och hypertoni

C) incidens och mortalitet i cancer D) hälsoekonomiska aspekter såsom

a. sjukvårdskonsumtion totalt och för olika diagnosgrupper

b. vårdkostnader

c. påverkan på indirekta kostnader (förlorade arbetsår och sjukfrånvaro)

d. interventionens kostnadseffektivitet (nettokostnaden för interventionen i relation till minskad hjärtkärlsjukdom och minskad dödlighet)

E) tidstrender av insjuknande och död i akut hjärtinfarkt, stroke, kardiovaskulär sjukdom samt totalmortalitet.

Studiedesign

Utvärdering av effekter på befolkningens hälsa av ett preventionsprogram av detta slag sker idealt genom en randomiserad kontrollerad studiedesign där individer slumpmässigt fördelas till intervention eller ej. Detta skedde inte i Sollentuna av uppenbara praktiska skäl. Av kritisk betydelse för tolkningen av resultat från föreliggande studie är att i avsaknad av en randomiserad design skapa jämförbarhet mellan de individer som kom att omfattas av interventionen på individ- eller populationsnivå och en jämförelsegrupp som inte omfattades av interventionen. I Sverige finns unika möjligheter att tillskapa en relevant jämförelsegrupp för föreliggande studie vilket är av stor betydelse för värdet av denna kohortstudie.

Studiepopulationer

1. Interventionsgruppen består av de personer (n=5940) som haft direktkontakt med preventionsprogrammet, d.v.s. genomgått en frivillig *opportunistisk* kardiovaskulär screening (frågeformulär, fysikalisk undersökning och provtagning i samband med besök på vårdcentralerna) mellan 1988-1993.
2. Anhöriga till individer som ingått i interventionsgruppen boende i Stockholms län under minst ett årsskifte under tiden för interventionen. Dessa identifieras med utnyttjande av Medicinskt Födelseregister och Flergenerationsregistret. Anhöriga definieras som föräldrar och adoptivföräldrar, biologiska och adoptivsyskon (både hel- och halvsyskon) och biologiska och adoptivbarn samt make/maka/sambo till de som ingår i interventionsgruppen.

3. Referenspopulation utgörs av samtliga individer som bott i Stockholms län någon gång under perioden 1988-93. Jämförelsegruppen identifieras med utnyttjande av sparade befolkningsregister per 31/12 varje år under den aktuella perioden.

Uppföljning i nationella register och databaser

Samtliga individer i de tre grupper som utgör studiepopulation kommer att följas upp avseende bl.a. sjukdomshistorik, socioekonomi och biokemiska riskfaktorer i nedan redovisade nationella register och forskningsdatabaser. När samtliga matchningar med

nationella register och AMORIS databasen är genomförda av registeransvariga på myndigheterna kommer endast avidentifierade och anonymiserade data att finnas tillgängliga för statistiska bearbetningar. Socialstyrelsen upprättar och spara nyckelfilen för att i framtiden kunna uppdatera med nya årgångar.

Socialstyrelsens hälsoregister:

- Patientregistret fr.o.m. 1964 (nationellt heltäckande avseende sluten vård fr.o.m. 1987och registrering av specialiserad öppen vård sedan 2001): variabler se bil 4.
- Dödsorsaksregistret fr.o.m. 1988: variabler se bil. 4
- Cancerregistret fr.o.m. 1958: variabler se bil. 4
- Medicinskt födelseregister (MFR) fr.o.m. 1973: variabler se bil.4
- Läkemedelsregistret fr.o.m. 2005: variabler se bil. 4

Statististiska centralbyråns register:

- Register över totalbefolkningen (RTB): variabler se bil. 5.1
- Flergenerationsregistret: variabler se bil. 5.2
- Folk och bostadsräkningar 1970, 1980, 1985 och 1990: variabler se bil. 5.3
- Utbildningsregistret (UREG): variabler se bil. 5.4 och ULF bil 5.5
- LISA: variabler se bil. 5.6

Försäkringskassans register:

- Mikrodata för analys av socialförsäkringen *(MIDAS)* variabler se bil 5.7

Karolinska Institutet

- Sollentuna prevention program (n=5940) databas. Variabler, se bil. 1-3.
- Apolipoprotein MOrtality RISk (AMORIS) databas. Variabler: se bil 6. Denna databas baseras på laboratorieanalyser från CALAB-laboratoriet under perioden 1986-1995 och omfattar ca 800,000 individer huvudsakligen från Stockholmsområdet. En dominerande del av proverna härrör från undersökningar i samband med företagshälsovård. Samtliga prover har analyserats på ett och samma laboratorium och med en väl dokumenterad metodik.

Utvärdering och analys

Effekten av interventionen kommer att utvärderas främst genom en jämförelse av risk för hjärt- kärlsjukdom och cancer under uppföljningstiden mellan interventionsgruppen respektive anhöriggruppen och befolkningen i Stockholms län vid tiden för interventionen. Detta kan ske genom att till interventionsgruppen matchade ”kontroller” väljs slumpmässigt ur befolkningen i Stockholms län med hänsyn till relevanta bakgrundsfaktorer (kön, ålder, boendeområde, socioekonomi, sjukdomshistorik, riskfaktorer). Alternativt eller komplementärt kan hela befolkningen utnyttjas som jämförelsegrupp och där jämförbarhet med interventionsgruppen skapas via multivariat metodik i analysen. Av avgörande betydelse för validiteten i denna ansats är tillgången till ett flertal väsentliga determinanter för kardiovaskulär sjukdom och cancer. I föreliggande studie är detta möjligt genom att utnyttja ett flertal nationella register samt AMORIS-databasen (se ovan).

Utöver den individbaserade jämförelsen kommer även sekulär trend i incidens och mortalitet i hjärt- kärlsjukdom i Sollentuna kommun jämfört med Stockholms län respektive riket för perioden 1980-2010 att analyseras.

Analyser kommer att göras med sedvanlig epidemiologisk metodik. Mortalitet och incidens under uppföljningstiden i interventions- respektive anhöriggruppen jämfört med befolkningen i länet kommer att analyseras univariat, med stratifierad analys samt med multivariat metodik utnyttjande i första hand Cox proportional hazards regression. Vid analyser av tidstrender i incidens används i första hand Poisson-regression. Slumpmässig osäkerhet beaktas genom beräkning av 95% konfidensintervall.

Den intervenerade kohorten utgörs av 5940 personer. Dessa följs i ca 20 års tid avseende sjuklighet, dödlighet och vårdkonsumtion. Incidensen i akut hjärtinfarkt i Stockholms län bland medelålders personer är i genomsnitt ca 4/1000 innevånare och år. Detta ger ett förväntat antal fall i denna studie omkring 500 fall enbart av detta utfall. Man kan räkna med över 300 fall av stroke under uppföljningstiden. Jämförelsegruppen kommer att utgöras av hela Stockholms län där ca 5000 hjärtinfarkter inträffar per år. Detta torde vara tillräckligt för en utvärdering med god precision av sjuklighet och dödlighet i hjärt-kärlsjukdom såväl i interna som externa jämförelser.

Hälsoekonomiska utfall kommer att studeras utifrån såväl ett samhällsperspektiv

(inkluderande alla identifierbara kostnader) som ett sjukvårdsperspektiv (inkluderande bara direkta kostnader). Interventions- och anhöriggruppernas kostnader kommer att jämföras med kontrollgruppen såväl över tid som kumulativt. Om interventionskostnaden överstiger eventuella besparingar, kommer en kostnads-effektanalys genomföras.

Svaghet och styrka

Interventionen i Sollentuna genomfördes inte som en randomiserad studie. Detta har för- och nackdelar. En fördel är att programmet utgör en intervention under 'normala' betingelser (klinisk vardag) och har hög grad av generaliserbarhet vad gäller praktiskt preventionsarbete. En nackdel är att jämförbarheten mellan de som deltog i interventionen och den övriga befolkningen kan vara begränsad. Som ovan nämnts kommer föreliggande studie ha goda möjligheter att kompensera för detta genom tillskapande av en relevant jämförelsegrupp och med tillgång till ett stort antal bakgrundsfaktorer. Tillgången till sparade befolkningsregister, nationella hälsoregister och register över socioekonomi samt AMORIS-databasen med uppgifter om biokemiska riskfaktorer skapar i själva verket internationellt sett unika möjligheter att genomföra denna utvärdering. Det kan dock inte uteslutas att en eventuellt observerad lägre dödlighet och sjuklighet till någon del kan bero på en selektion i den intervenerade gruppen. Studien är således mer deskriptiv jämfört med en randomiserad studie, men har å andra sidan hög grad av generaliserbarhet och en mycket lång uppföljningstid som är svår att upprätthålla i en randomiserad design.

Betydelse

Trots en gynnsam trend i såväl insjuknande som död i kardiovaskulära sjukdomar under de senaste decennierna är hjärt- och kärlsjukdomar fortfarande vårt största folkhälsoproblem. Vi vet idag att dessa sjukdomar går att förebygga och prevention har fått en mer central plats i hälso- och sjukvården. Nationella och internationella dokument fokuserar också allt mer på prevention, men vi behöver veta mera om hur detta kan genomföras i praktiken. Vilka lärdomar kan vi dra av genomförda preventionsprogram i svensk sjukvård? Har de avsedd effekt? Vetenskapliga utvärderingar av preventionsansatser inom hälso- och sjukvården vad gäller långtidseffekter på sjuklighet och dödlighet saknas fortfarande till stor del i Sverige och resultaten från våra planerade utvärderingar kan därför ha direkt praktisk betydelse för framtida preventionsarbete i

hälso- och sjukvården. Preventionsprogrammet i Sollentuna ger, i kombination med svenska nationella register goda möjligheter till en vetenskaplig utvärdering av primärprevention i ett långtidsperspektiv.

Ägare av databas

Karolinska Institutet äger databasen som är stationerad på Institutionen för medicin, Hjärtkliniken, Karolinska Universitetssjukhuset i Solna.

Styrkommitte

Ordförande och sammankallande: Professor Mai-Lis Hellenius,

Projektkoordinator Medicine doktor Gunilla Journath

Ledamot: Professor Ulf de Faire, professor Niklas Hammar, doc Ingvar Krakau, medicine doktor Peter Lindgren, docent Stig Elofsson, professor Göran Walldius

Ansökan till register

Se bilaga1 förteckning över variabler i respektive register.

Etikansökan diarienummer 1172-31

Registrering i databas

Studien kommer att registreras i www.clinicaltrials.gov samt databas på KI.

Referenser

1. Folkhälsorapport 2009. Sos 2009-126-71. Socialstyrelsen 2009.
2. http://www.socialstyrelsen.se/publikationer2011/2011-7-6
3. Hellénius M-L, Rosell M, Sandgren J, de Faire U. High prevalence of overweight and metabolic syndrome among 60 year old women and men in Stockholm, Sweden. Atherosclerosis 2000;151:276.

4. Berg C, Rosengren A, Aires N, Lappas G, Toren K, Thelle D, Lissner L. Trends in overweight and obesity from 1985 to 2002 in Goteborg, West Sweden. Int J Obes

2005;29:916-24.

5. Wilhelmsen L, Welin L, Svärdsudd K, Wedel H, Eriksson H, Hansson PO, Rosengren A. Secular changes in cardiovascular risk factors and attack rate of myocardial infarction among men aged 50 in Gothenburg, Sweden. Accurate prediction using risk models. J Intern Med. 2008;263:636-43.

6. Björck L, Rosengren A, Bennett K, Lappas G, Capewell S. Modelling the decreasing coronary heart disease mortality in Sweden between 1986 and 2002. Eur Heart J 2009;30:1046-56.

7. European Guidelines on CVD Prevention. www.escardio.org

8. Weinehall L, Hellsten G, Boman K, Hallmans G, Asplund K, Wall S. Can a sustainable community intervention reduce the health gap?--10-year evaluation of a Swedish community intervention program for the prevention of cardiovascular disease. Scand J Public Health Suppl 2001;56:59-68.

9. Lingfors H, Persson LG, Lindström K, Ljungquist B, Bengtsson C. Time for a "vision zero" concerning premature death from ischaemic heart disease? Scand J Prim Health Care 2002;20:28-32.

10. Hellénius M-L, de Faire U, Krakau I, Berglund B. Prevention of cardiovascular disease within the primary health care system. Feasibility of a prevention program within the Sollentuna Primary Health Catchment Area. Scand J Prim Health Care 1993;11:68-73.

11. Hellénius M-L, Johansson J, Elofsson S, de Faire U, Krakau I. Four years experience of a cardiovascular opportunistic screening and prevention programme in the primary health care in Sollentuna, Sweden. Scand J Prim Health Care 1999;17:111-5.

12. Johansson J, Hellénius M-L, Elofsson S, Krakau I. Self-report as a selection instrument in screening for cardiovascular disease risk. Am J Prev Med 1999;4:322-4.

13. Wersäll J, Krakau I, Hellénius M-L, Karlberg L. Hur uppfattar vårdcentralsbesökare screening av riskfaktorer för hjärt/kärlsjukdom? En studie från primärvården i Sollentuna. AllmänMedicin 1991;12:265-6.

14. Hellénius M-L, Nilsson P, Elofsson S, Johansson J, Krakau I. Reduction of high cholesterol levels associated with younger age and longer education in a primary health care programme for cardiovascular prevention. Scand J Prim Health Care 2005;23:75-81.

15. Hellénius M-L, de Faire U, Berglund B, Hamsten A, Krakau I. Diet and exercise are equally effective in reducing risk for cardiovascular disease. Results of a randomized controlled study in men with slightly to moderately raised cardiovascular risk factors. Atherosclerosis 1993;103:81-91.

16. Hellénius M-L, Brismar K, de Faire U, Berglund B. Effects on glucose tolerance and insulin secretion, Insulin-like Growth Factor-I and its binding protein IGFPB-1 in a randomized controlled diet and exercise study in healthy middle-aged men. J Intern Med 1995;238:121-30.

17. Hellénius M-L, Krakau I, de Faire U. Favourable long-term effects from advice on diet and exercise given to healthy men with raised cardiovascular risk factors. Nutr Metab Cardiovasc Dis 1997;7:293-300.

18. http://www.socialstyrelsen.se/NR/rdonlyres/2B5A6B25-2026-470C-A8BD-0E45AF95FAAA/13558/200912671.pdfik

Bilaga 1

Riskfaktorer variabler i SoPP

- Totalkolesterol
- LDL-kolesterol
- Triglycerider
- HDL-kolesterol
- F-blodglukos
- Systoliskt blodtryck
- Diastoliskt blodtryck
- rökvanor (cigg/dag)
- Längd
- Vikt
- BMI
- Stusskvot

Bilaga 2

Patientenkät baseline variabler i SoPP

- Högt blodtryck
- Förhöjda blodfetter
- Ansträngningsutlöst smärta i bröstet
- Ansträngningsutlöst vadsmärta
- Rökning
- Diabetes
- Övervikt
- Regelbunden motion
- Ärftlighet f hjärtkärlsjd (föräldrar el syskon före 60 års ålder hjärtbesvär/stroke, hypertoni, hyperkolesterolemi)
- Föräldrar avlidit före 60
- Annan risk f hjärtkärlsjd

Bilaga 3

Behandlingvariabler i SoPP

- Kostrådgivning
- Råd angående rökstopp
- EKG
- Remiss Korpen
- Motionsrådgivning
- Remiss rökavvänjningsgrupp
- Remiss överviktsgrupp
- Arbetsprov
- Läkemedel inte registrerat

Bilaga 4

Variabelförteckning

Uttag från Socialstyrelsens hälsoregister för samkörning med Sollentuna primärpreventionspopulation (SoPP) och AMORIS-databasen

I den databas som skapas skall information om ålder och kön finnas.

Patientregistret:

För varje individ önskas uppgift från samtliga vårdtillfällen, from 1964 avseende:
Kön, ålder, sjukhus, klinik, diagnoser), DRG-kod och DRG-vikt, yttre orsak till skada eller förgiftning, operationer, inskrivningsdatum, utskrivningsdatum och alla sjukhusinläggningar,

Dödsorsaksregistret:

Kön, ålder, dödsdatum, hemort (län, kommun, församling), underliggande dödsorsak, skadans natur, multipla dödsorsaker, dödsålder i år, dödsplats, mord, grund till dödsorsaken, arbetsolycka, skada/förgiftning, alkohol, narkotika, diabetes

Cancerregistret:
För varje individ önskas uppgift från samtliga diagnostillfällen from 1958 avseende:

Kön, ålder, sjukhus, klinik, diagnosdatum, tumörens lokalisation, morfologisk diagnos, tumörutbredning, grund för TNM, FIGO (sida, benignitet), patologi- och cytologiavdelning, diagnosgrund, obduktionsfynd, tumörnummer

Medicinskt födelseregister (MFR):
För varje individ önskas uppgift från samtliga graviditeter fr.o.m. 1973 enligt bilagd specifikation B.

Läkemedelsregistret:
För varje individ önskas uppgift från samtliga registreringstillfällen (uthämtade recept) from 2005 avseende:

Förskrivningsdatum, expeditionsdatum, ATC-kod och läkemedelskostnad.

Bilaga 5

Uttag av data från SCB för samkörning med Socialstyrelsens hälsoregister (bil 4) samt Sollentuna primärpreventionspopulation (SoPP) och AMORIS-databasen

Specifikation av önskade uppgifter från register vid SCB och MIDAS.

1) **Registret över totalbefolkningen (RTB)**:
   a) Personnummer (används för länkning)
   b) Län, kommun, församling
   c) Civilstånd
   d) Medborgarskap och födelseland
   e) Samhörighet och relationer
   f) Föräldrars födelseland
   g) Invandring (1969-) och utvandring (1961-)
2) **Flergenerationsregistret:** (Anhöriga: far, mor, syskon, barn)**.**
   a) Personnummer (används för länkning)
   b) Antal barn
   c) Tidpunkt för första barn
   d) Antal syskon
   e) Familjerelationer inom databasen
3) **Folk- och Bostadsräkningar** 1970, 1980, 1985 och 1990
   a) Civilstånd
   b) Medborgarskap och födelseland
   c) Sammanboende
   d) Utbildning
   e) Inkomst
   f) Socioekonomisk gruppering
   g) Förvärvsarbetets omfattning
   h) Anställningens art
   i) Yrke, yrkesställning och näringsgren
   j) Län, kommun, församling
   k) Tätortskod
4) **Undersökningar av levnadsförhållanden** (ULF)
   a) Hälsa och omsorg (1975/77, 1980-81, 1988-89, 1996-97, 2004-05):
      i) Bedömning av eget hälsotillstånd
      ii) Förekomst av långvarig sjukdom
      iii) Nedsatt syn eller hörsel
      iv) Nedsatt rörlighet
      v) Läkemedelsanvändning
      vi) Tandtillstånd och tuggförmåga
      vii) Rökvanor
      viii) Alkoholvanor

        ix) Matvanor
        x) Motionsvanor
        xi) Längd och vikt (BMI)
    b) Familj och sociala relationer
        i) Hushållets sammansättning
        ii) Har någon nära vän
    c) Ekonomi
        i) Arbetsinkomst för intervjuperson
        ii) Hushållets disponibla inkomst
    d) Sysselsättning och arbetstider
        i) Aktuell sysselsättning
        ii) Yrke
        iii) Näringsgren
        iv) Arbetslöshetserfarenhet
    e) Arbetsmiljö
        i) Fysisk arbetsmiljö
        ii) Psykosocial arbetsmiljö
    f) Bakgrundsvariabler
        i) Kön
        ii) Ålder
        iii) Civilstånd
        iv) Nationalitet
        v) Utbildningsnivå
        vi) Socioekonomisk grupp

5) **Befolkningens utbildning**
    a) Utbildning
    b) Högsta utbildning

6) **Longitudinell integrationsdatabas för sjukförsäkrings- och arbetsmarknadsstudier (LISA)**

    Uppgifter om utbildning, yrke, sysselsättning och inkomst longitudinellt.

7) **Mikrodata för analys av socialförsäkringen (MIDAS) tid: 1987-**

    Sjukersättning eller motsvarande a) Startdatum för sjukersättning, år, månad och dag
    b) Slutdatum för sjukersättning, år, månad och dag
    c) Orsak t sjukersättning, om angivet
    d) Sjukersättning som andel av heltid 0-100%
    e) Kostnad för sjukersättning om angivet

    Aktivitetsersättning eller motsvarande
    a) Startdatum för aktivitetsersättning, år, månad och dag
    b) Slutdatum för aktivitetsersättning, år, månad och dag
    c) Orsak t aktivitetsersättning, om angivet
    d) Aktivitetsersättning som andel av heltid 0-100%   e) Kostnad för aktivitetsersättning, om angivet.

Sjukpenning eller motsvarande

a) Startdatum för sjukersättning, år, månad och dag

b) Slutdatum för sjukersättning, år, månad och dag

c) Orsak t sjukersättning, om angivet

d) Sjukersättning som andel av heltid 0-100%

e) Kostnad för sjukersättning om angivet

Bilaga 6

AMORIS-registret variabler

Totalkolesterol
LDL-kolesterol
Triglycerider
HDL-kolesterol
ApoB
ApoA
ApoB/apoA
F-blodglukos
Längd
Vikt
BMI
