# Supplementary material for: Time Trends in Incidence and Mortality of Acute Myocardial Infarction, and All-Cause Mortality following a Cardiovascular Prevention Program in Sweden
Source: PLoS One. 2015 Nov 18;10(11):e0140201. doi: 10.1371/journal.pone.0140201 (PMC4651336; doi:10.1371/journal.pone.0140201)
Supplement: S3 Text — (PDF) [file pone.0140201.s013.pdf]

# Project plan
# Follow-up of Sollentuna prevention program (SoPP)

Gunilla Journath [1], Niklas Hammar [2,3], Ulf de Faire [2], Peter Lindgren[2], Ingvar Krakau[1], Stig Elofsson [4], Goran Walldius[2] and Mai-Lis Hellénius[1]

1. Department of Medicine, Karolinska Institutet, Karolinska University Hospital, Solna, Sweden
2. Institute of Environmental Medicine, Karolinska Institutet, Solna, Sweden
3. Patient Safety, Epidemiology, AstraZeneca R & D, Södertälje and Mölndal, Sweden
4. Stockholm University, Stockholm, Sweden

## Background

Although the incidence and mortality of cardiovascular diseases have declined in recent decades 41 percent of women and 39 percent of men died in 2010 in Sweden by these diseases [1,2].
The underlying factors are largely known [3-5]. Preventive interventions focusing on lifestyle change has been an increasingly central place in health care. Incidence and mortality rates have decreased. Treatment in health care has been estimated to account for 30-40%, and lifestyle changes for at least 50% [6]. Therefore, guidelines have increasingly focused on the importance of lifestyle changes [7]. Large-scale prevention programs have been implemented in Swedish primary care, e.g. in Västerbotten, Habo and Sollentuna [8-10]. However, evaluations of the long-term effects on cardiovascular health, as well as health economic evaluations, are still few.
Four health care centers, in Sollentuna Municipality, north of Stockholm, offered visitors, regardless of contact cause a health check with a focus on lifestyle and cardiovascular risk factors during the period 1988-92. A simple questionnaire was filled in (attachment 1) [11]. Height, weight, waist and hip measurements, blood pressure and blood lipids (fasting) was checked. In risk individuals fasting blood glucose levels were also checked (attachment 2). Individuals with elevated levels of risk factors were offered individual counselling with focus on lifestyle change by a physician and / or nurse (attachment 3). Individuals were given opportunity to participate in obesity-, smoking cessation- and physical activity groups. Close cooperation with physical activity organisation “Korpen” in Sollentuna started. Within a few years over 25 different custom exercise groups each semester were offered to patients who received a referral for physical activity by primary care staff (the predecessor of physical activity on prescription). One evening a week open lecture series for patients and families were offered during 17 years [10-12].

Before the start and during the first four years training for all staff in primary care were arranged at 40 different occasions. The training included basic scientific information and pedagogy of the lifestyle changes [1,3].

In parallel with the individualized work a large population-oriented work was conducted during the first 8 years. On 70 different occasions key people from grocery stores, day care centers, schools, municipalities, public dental care, associations, etc. were invited to information sessions and education on lifestyle and cardiovascular disease, and several collaborative projects were initiated [10-12].

## Declining risk factor levels in participants in the prevention program

During the first year, 2116 individuals were recorded in the program [10], and the majority (70%) had cardiovascular risk factors that required treatment and monitoring. An attitude survey among participants showed a positive attitude towards the prevention program [12]. A follow-up of the first four years (1988-1992) among 5622 persons registered in the program showed a high percentage of risk factors for cardiovascular disease. It turned out that those who initially had high cholesterol or high blood pressure significantly decreased their levels during a follow-up of in average 15 months [13]. Blood pressure fell by about 5 mm Hg in both men and women, and cholesterol levels by an average of 7% in men and 10% among women. A pronounced drop in triglycerides of 24% among men and 42% among women were observed [13]. Successful lowering of blood lipids have been shown to be associated with female gender and longer education [4].

## Effects of diet and exercise on cardiovascular risk

Participants in the prevention program have been used as a recruitment base to randomized controlled trials in which the effects of lifestyle intervention (mainly advice on diet and / or exercise) on cardiovascular risk factors and quality of life have been studied. A number of publications and three theses have been presented (A. Asplund-Carlsson 1994, ML Hellénius 1995, GK Naslund 1996). Low-intensity intervention on diet and / or physical activity in healthy middle-aged men with elevated cardiovascular risk has shown effects on weight, abdominal fat, blood pressure, lipids and insulin sensitivity after six months [15,16]. Several of the effects persisted at 18-month follow-up study [17]. Inflammatory markers and adhesion molecules were affected equally (Sjogren et al 2011). Factors that may affect the propensity to engage in diet and exercise programs, as well as adherence to a given intervention has also been studied.

## Overall objective

To evaluate long-term effects of an individual and population-oriented cardiovascular prevention program in primary care.

### Specific objectives

To study whether an individual and population-focused cardiovascular prevention program has affected those who participated in the prevention program, their families and the local population in a 20-year follow-up regarding:

A) incidence and mortality in acute myocardial infarction, stroke, cardiovascular disease (acute myocardial infarction, angina, stroke, heart failure and / or peripheral vascular disease), and total mortality.
B) drug treatment of diabetes, dyslipidemia and hypertension
C) incidence and mortality from cancer
D) health economic aspects such as
  a. the consumption of medical care overall and for different diagnostic groups
  b. the cost of care
  c. the impact on indirect costs (lost working years and sick leave)
  d. the intervention cost efficiency (net cost of the intervention in relation to reduced cardiovascular disease and reduced mortality)
E) time trends of incidence and death in acute myocardial infarction, stroke, cardiovascular disease and total mortality.

## Design

Evaluation of the effects on the population health of a prevention program of this kind is ideally done by a randomized controlled study design where individuals are randomly allocated to the intervention or not. This did not happen in Sollentuna, for obvious practical reasons. Of critical importance for the interpretation of the results from the present study is that in the absence of a randomized design for comparability between the individuals who were subject to intervention at the individual or population level and a comparison group who were not covered by the intervention. In Sweden, there are unique opportunities to create a relevant comparison group for this study, which is of great importance to the value of this cohort study.

## Study Populations

1. The intervention group consists of those individuals (n = 5940) who had direct contact with the prevention program, i.e. undergone a voluntary opportunistic cardiovascular screening (questionnaire, physical examination and testing in connection with visits to health centers) between 1988-1993.
2. Relatives of individuals included in the intervention group living in Stockholm County for at least one year-end during the time of the intervention. These are identified using the Medical Birth Registry and the Multi-Generation Register. Family members are defined as parents and adoptive parents, biological and adopted siblings (both full- and half-siblings) and biological and adopted children and husband / wife / partner to those in the intervention group.
3. The reference population consists of all individuals who lived in Stockholm County at some point during the period 1988-93. The comparison group is identified by use of saved population registers by 31/12 each year during the period.

## Follow-up in national registers and databases

The subjects in the study populations (1-3) will be followed on medical history, socio-economic and biochemical risk factors presented in research databases and National registers. When all the matches of national registers and research databases are done data will be available for statistical processing. All data will be anonymous. National Board of Health and Welfare will establish and save the key file in order to be able to update with new editions.

## The national Board of Health and Welfare:

• Patient Register 1964- (national comprehensive regarding patient care from 1987 and registration of specialized outpatient care since 2001): variables (appendix 4).
• Causes of Death 1988-: variables (appendix 4)
• Cancer register 1958-: variables (appendix 4)
• Medical Birth Register 1973-: variables (appendix 4)
• Drug Register 2005-: variables (appendix 4)

## Statististics Sweden:

• Total Population Register (TPR): variables (appendix 5.1)
• Multi-Generation Register: variables (appendix 5.2)
• People and housing censuses in 1970, 1980, 1985 and 1990: variables (appendix 5.3)
• Education Register (UREG): variables (appendix 5.4) and ULF (appendix 5.5)
• LISA variables (appendix 5.6)

## Social Insurance Agency:

• Micro data for the analysis of social insurance (MIDAS) variables (appendix 5.7)

## Karolinska Institutet

• Sollentuna prevention program (n = 5940) database. Variables (appendix 1-3).
• Apolipoprotein Mortality risk (AMORIS) database. Variables (appendix 6). This database is based on laboratory analyzes of CALAB Laboratory during the period from 1986 to 1995 and covers about 800,000 individuals, mainly from the Stockholm area. A dominant portion of the samples derived from studies related to occupational health. All samples were analyzed in the same laboratory using a well-documented methodology.

## Evaluation and statistical analysis

The effect of the intervention will be evaluated mainly by comparing the risk of cardiovascular disease and cancer during the follow-up period between the intervention group, the relative group and the population of Stockholm County at the time of the intervention. This can be done by making a matched control group from individuals in the rest of Stockholm County to the intervention group as "controls" randomly chosen from the population of Stockholm County with respect to relevant variables (gender, age, living area, socioeconomic status, medical history, risk factors). Alternatively or complementarily, the entire population is used as a comparison group and where comparability with the intervention group created via multivariate methods of analysis. Of critical importance to the validity of this approach is the availability of a number of important determinants of cardiovascular disease and cancer. In the present study, this is possible by taking advantage of several national records and the AMORIS database (see above).
In addition to the individual-based comparison secular trends in incidence and mortality from cardiovascular disease in Sollentuna, compared to Stockholm County for the period 1980-2010 will be analyzed.
Analyses will be done with standard epidemiological methods. Mortality and incidence during the follow-up period in the intervention or relative group compared to the population of the county will be analyzed in univariate, with stratified analysis and multivariate methods primarily using Cox proportional hazards regression. In analyzes of time trends in incidence Poisson regression is primarily used. Random uncertainties are taken into account by calculating the 95% confidence intervals.

The intervened cohort consists of 5940 people. These are followed for about 20 years for morbidity, mortality and health care consumption. The incidence of acute myocardial infarction in Stockholm County among middle-aged people are averaging about 4/1000 inhabitants and year. This gives about 500 expected numbers of cases in this study, solely by this outcome. More than 300 cases of stroke are expected during the follow-up. The comparison group will consist of the entire Stockholm County, where about 5,000 heart attacks occur annually. This should be sufficient for an evaluation with good precision of morbidity and mortality from cardiovascular disease in both internal and external comparisons.
Health economic outcomes will be studied from both a societal perspective (including all identifiable costs) as a healthcare perspective (including only direct costs). The intervention and relative groups' costs will be compared with the control group, both over time and

cumulatively. If intervention costs exceed any savings, a cost-effectiveness analysis will be conducted.

## Weakness and strength

Intervention in Sollentuna was not performed as a randomized trial. This has advantages and disadvantages. One advantage is that the program is an intervention under ‘normal’ conditions (everyday clinical practice) and has a high degree of generalizability with regard to practical prevention efforts. One drawback is that the comparability of those who participated in the intervention and the general population may be limited. As mentioned above, the present study has good opportunities to compensate for this by the creation of a relevant comparison group and with access to a large number of background factors. Access to stored vital records, national health registers and records of socioeconomics and AMORIS database with data on biochemical risk factors creates in fact internationally unique opportunities to conduct this evaluation. It cannot be ruled out that any observed lower mortality and morbidity in any part may be due to a selection in the intervened group. The study is thus more descriptive compared to a randomized study, but on the other hand have a high degree of generalization and a very long follow-up that is difficult to maintain in a randomized design.

## Interpretation

Despite favorable trends in both incidence and mortality from cardiovascular disease in recent decades cardiovascular diseases are still our biggest public health problem. We know today that these diseases are preventable and prevention has been given a more central place in health care. National and international documents also focus increasingly on prevention, but we need to know more about how this can be implemented in practice. What lessons can we draw from implemented prevention programs in Swedish health care? Do they have the intended effect? Scientific evaluations of prevention approaches in health care in terms of long-term effects on morbidity and mortality is still missing to a large extent in Sweden and the results of our planned evaluations may therefore have direct practical implications for future prevention work in health care. The Prevention Program in Sollentuna provides, in combination with the Swedish national registers, good opportunities for a scientific evaluation of primary prevention in the long term.

## Owner of the database

Karolinska Institutet own the database which is kept at the Department of Medicine, Department of Cardiology, Karolinska University Hospital in Solna.

## Steering committee

Chairman and Convener: Professor Mai-Lis Hellenius,
Project Coordinator: PhD Gunilla Journath
Director: Professor Ulf de Faire, Professor Niklas Hammar, doc Ingvar Krakau, MD Peter Lindgren, Associate Professor Stig Elofsson, Professor Göran Walldius

**Application to register**
See Attachment 1 list of variables in the respective registers.
**Ethics Application** reference number 1172-31

The study will be registered in www.clinicaltrials.gov and a database on KI.

## References

1. Public Health Report 2009. Sos 2009-126-71. The National Board of Health and Welfare in 2009.

2. Causes of Death 2010. http://www.socialstyrelsen.se/publikationer2011/2011-7-6. Accessed August 25, 2015.

3. Hellénius ML, Rosell M, Sandgren J, de Faire U. High prevalence of overweight and metabolic syndrome among 60 year old women and men in Stockholm, Sweden. Atherosclerosis 2000; 151: 276.

4. Berg C, Rosengren A, Aires N, Lappas G, Toren K, Thelle D, Lissner L. Trends in overweight and obesity from 1985 to 2002 in Goteborg, West Sweden. Int J Obes 2005; 29: 916-24.

5. Wilhelmsen L, Welin L, Svärdsudd K, Wedel H, Eriksson H, Hansson PO, Rosengren A. Secular changes in cardiovascular risk factors and attack rate of myocardial infarction among men aged 50 in Gothenburg, Sweden. Accurate prediction using risk models. J Intern Med 2008; 263: 636-43.

6. Bjorck L, Rosengren A, Bennett K, Lappas G, Capewell S. Modelling the decreasing coronary heart disease mortality in Sweden between 1986 and 2002. Eur Heart J 2009; 30: 1046-56.

7. The European Guidelines on CVD Prevention. www.escardio.org. Accessed August 25, 2015.

8. Weinehall L, Hellsten G, Boman K, Hallmans G, Asplund K, Wall S. Can a sustainable community intervention reduce the health gap? - 10-year evaluation of a Swedish community intervention program for the prevention of cardiovascular disease. Scand J Public Health Suppl 2001; 56: 59-68.

9. Lingfors H, Persson LG, Lindstrom K, Ljungquist B, Bengtsson C. Time for a "vision zero" concerning premature death from ischemic heart disease? Scand J Prim Health Care 2002; 20: 28-32.

10. Hellénius ML, de Faire U, Krakau I, Berglund B. Prevention of cardiovascular disease within the primary health care system. Feasibility of a prevention program within the Sollentuna Primary Health Catchment Area. Scand J Prim Health Care 1993; 11: 68-73.

11. Hellénius ML, Johansson J, Elofsson S, de Faire U, Krakau I. Four years experience of a cardiovascular opportunistic screening and prevention programme in the primary health care in Sollentuna, Sweden. Scand J Prim Health Care 1999; 17: 111-5.

12. Johansson J, Hellénius ML, Elofsson S, Krakau I. Self-report as a selection instrument in screening for cardiovascular disease risk. Am J Prev 1999; 4: 322-4.

13. Wersäll J, Krakau I, Hellénius ML, Karlberg L. How does the health care center visitor appreciate screening of risk factors for cardiovascular disease? A study from primary care in

Sollentuna. General Practice 1991; 12: 265-6. (In Swedish)

14. Hellénius ML, Nilsson P, Elofsson S, Johansson J, Krakau I. Reduction of high cholesterol levels associated with younger age and longer education in a primary health care program for cardiovascular prevention. Scand J Prim Health Care 2005; 23: 75-81.

15. Hellénius ML, de Faire U, Berglund B, Hamsten A, Krakau I. Diet and exercise are equally effective in reducing risk for cardiovascular disease. Results of a randomized controlled study in men with slightly to moderately raised cardiovascular risk factors. Atherosclerosis 1993; 103: 81-91.

16. Hellénius ML, Brismar K, de Faire U, Berglund B. Effect on glucose tolerance and insulin secretion, Insulin-like Growth Factor-I and Its binding protein IGFBP-1 in a randomized controlled diet and exercise study in healthy middle-aged men. J Intern Med 1995; 238: 121-30.

17. Hellénius ML, Krakau I, de Faire U. Favourable long-term effects from advice on diet and exercise given to healthy men with raised cardiovascular risk factors. Nutr Metab Cardiovasc Dis 1997; 7: 293-300.

Appendix 1

**Risk factor variables in SoPP**

• Total cholesterol
• LDL cholesterol
• Triglycerides
• HDL cholesterol
• F-blood glucose
• Systolic blood pressure
• Diastolic blood pressure
• Smoking habits (cig / day)
• Length
• Weight
• BMI
• Waist/Hip ratio

Appendix 2

**Patient Survey - baseline variables in SoPP**

• High blood pressure
• Elevated blood lipids
• Exercise-induced chest pain
• Exercise-induced pain in leg
• Smoking
• Diabetes
• Overweight
• Regular exercise
• Hereditary cardiovascular disease (parents or siblings with heart disease / stroke before age 60, hypertension, hypercholesterolemia)
• Parents died before 60 years of age
• Other risk factors for cardiovascular disease

Appendix 3

**Treatment Variables SoPP**

• Dietary advice
• Advice regarding smoking cessation
• ECG
• Referral physical activity
• Exercise advice
• Referral smoking cessation group
• Referral obesity group
• Work capacity test
• Medications not registered

Appendix 4

**List of variables**

**Extracts from registries at the National Board of Health and Welfare for matching with the Sollentuna primary prevention population (SoPP) and the AMORIS database**

Age and sex should be included in all databases

**Patient Register:**
For each individual all admissions from 1964 regarding:
Gender, age, hospital, clinic, diagnoses, DRG code and DRG weight, external cause of injury or poisoning, operations, enrollment date, discharge date, and all hospital admissions.
NOTE treatment in hospitals and outpatient ward in hospitals (specialist).

**Causes of Death register:**
Gender, age, date of death, place of residence (county, municipality, parish), the underlying cause of death, nature of the damage, multiple causes of death, the death age in years, the place of death, murder, cause of death, working accident, injury / poisoning, alcohol, drugs, diabetes

**Cancer register:**
For every individual all cancer diagnosis from 1958 regarding:
Gender, age, hospital, clinic, date of diagnosis, tumor localization, morphologic diagnosis, tumor extent, due to the TNM, FIGO (position, benign), pathology and cytology department, diagnostic basis, autopsy findings, tumor number.

**Medical Birth Register (MFR):**
For every individual all child births from 1973.

**Drug Register:**
For every individual all registration occasions (collected recipes) from 2005 and onwards regarding: Prescription date, expedition dates, ATC code and drug expenses.

Appendix 5

# Collection of data from Statistics Sweden for linking with the National Board of Health and Welfare records (appendix 4) and Sollentuna primary prevention population (SoPP) and the AMORIS database

Specification of the desired data from the registers at Statistics Sweden and the MIDAS.

**1) Total Population Register (TPR):**
- a) Personal identity number (used for linking)
- b) County, municipality, parish
- c) Marital status
- d) Citizenship and country of birth
- e) Affinity and relationships
- f) Parents' country of birth
- g) Immigration (1969-) and emigration (1961-)

**2) Multi-Generation Register (Family members: father, mother, siblings, children).**
- a) Personal identity number (used for linking)
- b) Number of children
- c) Date of birth of first child
- d) Number of siblings
- e) Family relationships in the database

**3) Population and housing censuses in 1970, 1980, 1985 and 1990**
- a) Marital status
- b) Citizenship and country of birth
- c) Cohabiting
- d) Training
- e) Income
- f) Socio-economic grouping
- g) Acquisition Scope of Work
- h) The nature of the employment
- i) Occupation, occupational status and industry
- j) County, municipality, parish
- k) Urban center code

**4) Surveys of Living Conditions (SLC)**
- a) Health Care (1975/77, 1980-81, 1988-89, 1996-97, 2004-05):
  - i) Assessment of own health
  - ii) The presence of long-term illness
  - iii) Impaired vision or hearing
  - iv) Reduced mobility
  - v) Medication
  - vi) Dental status and chewing ability
  - vii) Smoking habits
  - viii) Drinking habits
  - ix) Eating Habits
  - x) Exercise Habits
  - xi) Height and weight (BMI)
- b) Family and social relationships
  - i) Household composition
  - ii) Have a close friend

c) Economy
  i) Earned income of respondent
  ii) Household disposable income
d) Employment and working hours
  i) Current Employment
  ii) Job title
  iii) Industry
  iv) Unemployment Experience
e) Work environment
  i) Physical environment
  ii) Psychosocial work environment
f) Background variables
  i) Gender
  ii) Age
  iii) Marital status
  iv) Nationality
  v) Education
  vi) Socio-economic group

**5) Population Education**

a) Training
b) Highest education

**6) Longitudinal integration database for health insurance and labor market studies (LISA)**

Longitudinal data on education, occupation, employment and income.

**7) Micro data for the analysis of social insurance (MIDAS)**

Time: 1987-, Sickness benefits or equivalent
a) Start date of sickness period, year, month and day
b) Closing date for sickness period, year, month and day
c) Cause of sickness benefits, if specified
d) Sickness benefits as a percentage of full-time 0-100%
e) Cost of sickness benefit if specified

Activity benefits or equivalent
a) Start date of the activity period, year, month and day
b) Closing date for activity period, year, month and day
c) Cause of activity period, if indicated
d) Activity as a percentage of full-time 0-100%
e) Cost of activity benefits, if indicated.

Sickness pay or equivalent
a) Start date of sickness period, year, month and day
b) Closing date for sickness period, year, month and day
c) Cause for sickness benefits, if specified
d) Sickness benefits as a percentage of full-time 0-100%
e) Cost of sickness benefit if specified

Appendix 6

**AMORIS registry variables**

Total cholesterol
LDL cholesterol
Triglycerides
HDL cholesterol
ApoB
ApoA
ApoB / apoA
F-blood glucose
Length
Weight
BMI
